# Supplementary material for: Methodological aspects of economic evaluations conducted in the palliative or end of life care settings: a systematic review protocol
Source: BMJ Open. 2020 May 27;10(5):e035760. doi: 10.1136/bmjopen-2019-035760 (PMC7259853; doi:10.1136/bmjopen-2019-035760)
Supplement: Supplementary data [file bmjopen-2019-035760supp001.pdf]

APPENDIX

Appendix 1

PRISMA-P (Preferred Reporting Items for Systematic Review and Meta-Analysis Protocols) 2015 checklist: recommended items to address in a systematic review protocol*

Section and topic	Item No	Checklist item	Reported
**ADMINISTRATIVE INFORMATION**			
Title:			
Identification	1a	Identify the report as a protocol of a systematic review	Title page
Update	1b	If the protocol is for an update of a previous systematic review, identify as such	N/A
Registration	2	If registered, provide the name of the registry (such as PROSPERO) and registration number	Page 2
Authors:			
Contact	3a	Provide name, institutional affiliation, e-mail address of all protocol authors; provide physical mailing address of corresponding author	Title page
Contributions	3b	Describe contributions of protocol authors and identify the guarantor of the review	Page 10
Amendments	4	If the protocol represents an amendment of a previously completed or published protocol, identify as such and list changes; otherwise, state plan for documenting important protocol amendments	N/A
Support:			
Sources	5a	Indicate sources of financial or other support for the review	Page 10
Sponsor	5b	Provide name for the review funder and/or sponsor	Page 10
Role of sponsor or funder	5c	Describe roles of funder(s), sponsor(s), and/or institution(s), if any, in developing the protocol	Page 10
**INTRODUCTION**			
Rationale	6	Describe the rationale for the review in the context of what is already known	Page 3
Objectives	7	Provide an explicit statement of the question(s) the review will address with reference to participants, interventions, comparators, and outcomes (PICO)	Page 3-4
**METHODS**			
Eligibility criteria	8	Specify the study characteristics (such as PICO, study design, setting, time frame) and report characteristics (such as years considered, language, publication status) to be used as criteria for eligibility for the review	Page 4-6

Information sources	9	Describe all intended information sources (such as electronic databases, contact with study authors, trial registers or other grey literature sources) with planned dates of coverage	Page 5
Search strategy	10	Present draft of search strategy to be used for at least one electronic database, including planned limits, such that it could be repeated	Page 6-7 Appendix 2
Study records:			
Data management	11a	Describe the mechanism(s) that will be used to manage records and data throughout the review	Page 6
Selection process	11b	State the process that will be used for selecting studies (such as two independent reviewers) through each phase of the review (that is, screening, eligibility and inclusion in meta-analysis)	Page 7-8
Data collection process	11c	Describe planned method of extracting data from reports (such as piloting forms, done independently, in duplicate), any processes for obtaining and confirming data from investigators	Page 8
Data items	12	List and define all variables for which data will be sought (such as PICO items, funding sources), any pre-planned data assumptions and simplifications	Page 4-6
Outcomes and prioritization	13	List and define all outcomes for which data will be sought, including prioritization of main and additional outcomes, with rationale	Page 4 and 6
Risk of bias in individual studies	14	Describe anticipated methods for assessing risk of bias of individual studies, including whether this will be done at the outcome or study level, or both; state how this information will be used in data synthesis	Page 9
Data synthesis	15a	Describe criteria under which study data will be quantitatively synthesised	Page 8
	15b	If data are appropriate for quantitative synthesis, describe planned summary measures, methods of handling data and methods of combining data from studies, including any planned exploration of consistency (such as I^2, Kendall's τ)	N/A
	15c	Describe any proposed additional analyses (such as sensitivity or subgroup analyses, meta-regression)	N/A
	15d	If quantitative synthesis is not appropriate, describe the type of summary planned	N/A
Meta-bias(es)	16	Specify any planned assessment of meta-bias(es) (such as publication bias across studies, selective reporting within studies)	N/A
Confidence in cumulative evidence	17	Describe how the strength of the body of evidence will be assessed (such as GRADE)	Page 9

* It is strongly recommended that this checklist be read in conjunction with the PRISMA-P Explanation and Elaboration (cite when available) for important clarification on the items. Amendments to a review protocol should be tracked and dated. The copyright for PRISMA-P (including checklist) is held by the PRISMA-P Group and is distributed under a Creative Commons Attribution Licence 4.0.

The PRISMA for Abstracts Checklist

TITLE	CHECKLIST ITEM	REPORTED ON PAGE #
**1.Title**	Identify the report as a systematic review, meta-analysis, or both.	2 (Introduction)
**BACKGROUND**		
**2.Objectives:**	The research question including components such as participants, interventions, comparators, and outcomes.	2 (Introduction)
**METHODS**		
**3.Eligibility criteria:**	Study and report characteristics used as criteria for inclusion.	2 (Introduction)
**4.Information sources:**	Key databases searched and search dates.	2 (Methods and analysis)
**5. Risk of bias:**	Methods of assessing risk of bias.	2 (Methods and analysis)
**RESULTS**		
**6. Included studies:**	Number and type of included studies and participants and relevant characteristics of studies.	n.a.
**7. Synthesis of results:**	Results for main outcomes (benefits and harms), preferably indicating the number of studies and participants for each. If meta-analysis was done, include summary measures and confidence intervals.	n.a.
**8. Description of the effect:**	Direction of the effect (i.e. which group is favoured) and size of the effect in terms meaningful to clinicians and patients.	n.a.
**DISCUSSION**		
**9. Strengths and Limitations of evidence:**	Brief summary of strengths and limitations of evidence (e.g. inconsistency, imprecision, indirectness, or risk of bias, other supporting or conflicting evidence)	n.a.
**10. Interpretation:**	General interpretation of the results and important implications	n.a.
**OTHER**		
**11. Funding:**	Primary source of funding for the review.	2 (Acknowledgements)
**12. Registration:**	Registration number and registry name.	2 (Methods and analysis)

Appendix 2

Search syntax in different databases

EMBASE.com

#33 #28 NOT #31 AND [1-1-1999]/sd NOT [15-11-2019]

#32 #28 NOT #31

#31 #29 NOT #30

#30 #29 AND ([young adult]/lim OR [adult]/lim OR [middle aged]/lim OR [aged]/lim OR [very elderly]/lim)

#29 #28 AND ([adolescent]/lim OR [child]/lim OR [fetus]/lim OR [infant]/lim OR [newborn]/lim OR [preschool]/lim OR [school]/lim)

#23 NOT #26 AND ([dutch]/lim OR [english]/lim OR [french]/lim OR [german]/lim OR

#28 [spanish]/lim)

#27 #23 NOT #26

#26 #24 OR #25

#25 waste:jt OR 'life cycle assessment':jt

#24 recycl*:ti,de,kw,jt

#23 #13 AND #22

#22 #14 OR #15 OR #16 OR #17 OR #18 OR #19 OR #20 OR #21

#21 (('health care' OR healthcare) NEAR/3 cost*):ti,kw

#20 economics:ti,kw

(economic* NEAR/3 (evaluat* OR aspect* OR health OR analy* OR model* OR framework* OR

#19 'frame work*' OR method*)):ti,ab,kw

#18 'health care cost'/exp/mj

#17 'economics'/mj

#16 'economic model'/de

#15 'health economics'/de

#14 'economic evaluation'/de

#13 #1 OR #2 OR #3 OR #4 OR #5 OR #6 OR #7 OR #8 OR #9 OR #10 OR #11 OR #12

#12 bereave*:de,ti,ab,kw

#11 hospice*:de,ti,ab,kw

#10 'advanced cancer':ti,ab,kw

#9 'end of life':de,ti,ab,kw OR 'last year of life':de,ti,ab,kw OR 'lyol':de,ti,ab,kw OR 'life s end':de,ti,ab,kw

#8 (terminal* NEAR/6 (care* OR caring OR ill OR illness* OR patient*)):ti,ab,kw

#7 palliat*:ti,ab,kw

#6 'hospice'/de

#5 'terminal disease'/de

#4 'palliative therapy'/exp

#3 'terminal care'/exp

#2 'terminally ill patient'/exp

#1 'palliative nursing'/de

EBM Reviews - Health Technology Assessment Database (OVID) (discontinued at the end of 2016)

1 Palliative Care/

2 exp Terminal Care/

3 terminally ill/

4 palliat*.mp.

5 (terminal* adj6 (care or caring or ill or illness*)).mp.

6 (end of life or last year of life or lyol or life* end).mp.

7 advanced cancer.mp.

8 hospices/

9 hospice*.mp.

10 bereave*.mp.

11 1 or 2 or 3 or 4 or 5 or 6 or 7 or 8 or 9 or 10

12 exp Health Care Costs/

13 ((health care or healthcare) adj3 cost*).mp.

14 "Costs and Cost Analysis"/

15 Cost-Benefit Analysis/mt [Methods]

16 exp models, economic/

17 (economic* adj3 (evaluat* or aspect* or health or analy* or model* or framework* or frame work* or method*)).mp.

18 economics.mp.

19 12 or 13 or 14 or 15 or 16 or 17 or 18

20 11 and 19

21 limit 20 to "all child (0 to 18 years)"

22 limit 20 to (dutch or english or german or spanish or french)

EBM Reviews - NHS Economic Evaluation Database (OVID) (discontinued at the 31st March 2015)

1 Palliative Care/

2 exp Terminal Care/

3 terminally ill/

4 palliat*.mp. [mp=title, text, subject heading word]

5 (terminal* adj6 (care or caring or ill or illness*)).mp. [mp=title, text, subject heading word]

6 (end of life or last year of life or lyol or life* end).mp. [mp=title, text, subject heading word]

7 advanced cancer.mp. [mp=title, text, subject heading word]

8 hospices/

9 hospice*.mp. [mp=title, text, subject heading word]

10 bereave*.mp. [mp=title, text, subject heading word]

11 1 or 2 or 3 or 4 or 5 or 6 or 7 or 8 or 9 or 10

12	limit 11 to "all child (0 to 18 years)"
13	limit 12 to "all adult (19 plus years)"
14	12 not 13
15	11 not 14
16	limit 15 to yr="1999 - 2015"

Appendix 3

Data extraction form: methodological studies

**Reviewer:**	………………………………	**Date:**	………………………………
**Author:**	………………………………	**Country:**	………………………………
**Reference type:**	………………………………	**Manuscript code:**	………………………………
**Name of journal/book chapter/website etc. :**	………………………………	**Language of publication**	………………………………
**Date of publication:**	………………………………	**Page number:**	………………………………
**Study design:**			
Case report	O	Review (scoping, systematic, narrative)	O
Economic evaluation	O	Guideline or checklist	O
Editorial/commentary/letter:	O	Other	O
Cohort	O		
**Quality appraisal score:**			
Quality appraisal applied:	………………………………	Quality rating:	………………………………
**Aspect of economic evaluation (EE) described:**			
O Study population	O Study perspective	O Time horizon	O Type of EE
O Outcomes	O Data (sources)	O Costs	O Resource use measurement
O Valuation method	O Adjustments for inflation/deflation	O Discounting	O Ethical implications
O Decision modeling	O Care setting	O Other: ………………………………………	

Data extraction form: applied economic evaluations

**Reviewer:**	………………………………	**Date:**	………………………………
**Author:**	………………………………	**Manuscript code:**	………………………………
**Name of journal:**	………………………………	**Language of publication**	………………………………
**Date of publication:**	………………………………		
**Study characteristics:**			
Funding source (p.n.*)	………………………………	Year of analysis (p.n.):	………………………………
Study design (p.n.):	………………………………	Type of economic evaluation (CEA, CUA., etc) (p.n.):	………………………………
Author stated analytical perspective (p.n.):	………………………………	Time frame of analysis (p.n.):	………………………………
Country (p.n.):	………………………………	Study sector (hospice, home, hospital, etc. (p.n.):	………………………………
Modeling used (p.n.):	………………………………	Quality appraisal score (Quality appraisal):	………………………………
Intervention description:	………………………………		
**Patient cohort characteristics:**			
Control group size:	………………………………	Intervention group size:	………………………………
Target population definiton:	………………………………		
**Methods :**			
Analysis of uncertainty:	………………………………		
**Outcomes :**			
Outcomes measured:	………………………………	Discounting:	………………………………
Data sources :	………………………………		
**Costs:**			
Cost categories included:	………………………………	Discounting:	………………………………

Unit cost sources:	……………………………….	Resource use info source:	……………………………….
Reviewer stated analytical perspective:	……………………………….	Year of costing:	……………………………….
Adjustments for inflation/deflation:	……………………………….		
**Cost-effectiveness:**			
Main CE results:	……………………………….	Analysis of uncertainty:	……………………………….

*p.n. - page number
