# Supplementary material for: Methodological aspects of economic evaluations conducted in the palliative or end of life care settings: a systematic review protocol
Source: BMJ Open. 2020 May 27;10(5):e035760. doi: 10.1136/bmjopen-2019-035760 (PMC7259853; doi:10.1136/bmjopen-2019-035760)
Supplement: Author's manuscript [file bmjopen-2019-035760.draft_revisions.pdf]

BMJ Open is committed to open peer review. As part of this commitment we make the peer review history of every article we publish publicly available.

When an article is published we post the peer reviewers' comments and the authors' responses online. We also post the versions of the paper that were used during peer review. These are the versions that the peer review comments apply to.

The versions of the paper that follow are the versions that were submitted during the peer review process. They are not the versions of record or the final published versions. They should not be cited or distributed as the published version of this manuscript.

BMJ Open is an open access journal and the full, final, typeset and author-corrected version of record of the manuscript is available on our site with no access controls, subscription charges or pay-per-view fees (http://bmjopen.bmj.com).

If you have any questions on BMJ Open's open peer review process please email info.bmjopen@bmj.com

BMJ Open

Methodologic aspects of economic evaluations conducted in the palliative or end-of-life-care settings: a systematic review protocol

Journal:	*BMJ Open*
Manuscript ID	bmjopen-2019-035760
Article Type:	Protocol
Date Submitted by the Author:	14-Nov-2019
Complete List of Authors:	Fischer, Claudia; Medical University of Vienna Center for Public Health, Health Economics<br>Chwala, Eva; Medical University of Vienna, University Library<br>Simon, Judith; Medical University of Vienna,
Keywords:	PALLIATIVE CARE, STATISTICS & RESEARCH METHODS, HEALTH ECONOMICS

SCHOLARONE™
Manuscripts

I, the Submitting Author has the right to grant and does grant on behalf of all authors of the Work (as defined in the below author licence), an exclusive licence and/or a non-exclusive licence for contributions from authors who are: i) UK Crown employees; ii) where BMJ has agreed a CC-BY licence shall apply, and/or iii) in accordance with the terms applicable for US Federal Government officers or employees acting as part of their official duties; on a worldwide, perpetual, irrevocable, royalty-free basis to BMJ Publishing Group Ltd ("BMJ") its licensees and where the relevant Journal is co-owned by BMJ to the co-owners of the Journal, to publish the Work in this journal and any other BMJ products and to exploit all rights, as set out in our licence.

The Submitting Author accepts and understands that any supply made under these terms is made by BMJ to the Submitting Author unless you are acting as an employee on behalf of your employer or a postgraduate student of an affiliated institution which is paying any applicable article publishing charge ("APC") for Open Access articles. Where the Submitting Author wishes to make the Work available on an Open Access basis (and intends to pay the relevant APC), the terms of reuse of such Open Access shall be governed by a Creative Commons licence – details of these licences and which Creative Commons licence will apply to this Work are set out in our licence referred to above.

Other than as permitted in any relevant BMJ Author's Self Archiving Policies, I confirm this Work has not been accepted for publication elsewhere, is not being considered for publication elsewhere and does not duplicate material already published. I confirm all authors consent to publication of this Work and authorise the granting of this licence.

For peer review only

Methodologic aspects of economic evaluations conducted in the palliative or end-of-life-care settings: a systematic review protocol

Claudia Fischer[1], Eva Chwala[2], Judit Simon[1]

Corresponding author: Claudia Fischer[1]

For peer review only

ABSTRACT

Introduction:

Since the number of patients in advanced stages of incurable conditions is rising, the role of palliative care is also increasing. In light of this increasing patient population, as well as new (expensive) drugs and treatments, quality research providing evidence for decision making is required. However, common research guidance is lacking in this field, especially in respect to the methods applied in economic evaluations.

Methods and analysis:

A systematic review will be conducted using MEDLINE, EMBASE, Health Technology Assessment Database as well as NHS Economic Evaluation Database to identify studies published from 1999 onwards with relevant information on methodological challenges, potential solutions and recommendations for conducting economic evaluations in the palliative and end-of-life care settings. Two independent reviewers will conduct two screening rounds; any discrepancies will be resolved by discussion and consensus. Data will be extracted using a pre-designed data extraction form and consequently narratively synthesized and categorized depending on the methodological aspect they are addressing.

Ethics and dissemination:

Ethical approval is not required, as this is a planned systematic review of published literature. An article reporting on the results of the systematic review will be disseminated in a related peer-reviewed journal, as well as presented at leading palliative care and health economic conferences.

For peer review only

For peer review only - http://bmjopen.bmj.com/site/about/guidelines.xhtml

STRENGTHS AND LIMITATIONS OF THIS STUDY

- To our knowledge, this will be the first systematic review to identify methodological aspects and challenges to consider when conducting an economic evaluation in the palliative and end-of-life care setting.
- The inclusive search strategy of this systematic review includes four electronic databases of peer-reviewed literature and tailored search strings, carefully refined for every database searched in cooperation with an information specialist.
- The systematic review protocol is reported according to the recommendations of the Preferred Reporting Items for Systematic Review and Meta-Analysis Protocols (PRISMA-P) statement. Registration with the International Prospective Register of Systematic Reviews (PROSPERO) is ongoing.
- Articles published in English, German, Dutch, French and Spanish will be considered as eligible for inclusion.

For peer review only

INTRODUCTION

Over the past century, patterns of mortality in the Western world have changed [1]. While sudden death, mainly due to infectious disease, used to be the major cause of death, nowadays there is an increase of the ageing population living with advanced stages of incurable chronic conditions such as cancer, chronic heart failure or chronic obstructive pulmonary disease (COPD) near the end of life [2, 3].

It is crucial to enable this growing patient group to live well at the end of their lives as well as to die well [4, 5]. This requires quality research providing evidence for decisions on clinical guidelines, treatments and services [6]. In light of the new (expensive) drugs and interventions, an effective and efficient resource allocation is of great importance in the palliative and end-of-life care setting [7]. Studies have estimated the cost of care in the last year of life at 25 to 30% of all medical expenditures during a lifetime [8, 9]. This number is thought to even further increase in light of the ageing population and the prospect of potentially reduced number of informal carers in the future [10].

Economic evaluations are the comparative analysis of alternative courses of action in terms of both, their costs and consequences [11], and have been widely applied in other fields to answer questions in regards to cost-effectiveness of health care interventions and inform decision-makers and commissioners [12]. Nevertheless, the economic component has often been overlooked in palliative care studies, leaving a number of unanswered questions for the quality assurance, monitoring, funding, and evaluation of palliative care [13, 14]. End-of-life care research lacks common research guidance [15], which would be essential as the palliative care setting differs from any other health care field in several aspects i.e. patient population, ultimate aim of the interventions (quality of dying as opposed to quality of living) and relevant outcome measures. In order to generate valid und useful evidence and decision base, the research methods of economic evaluations applied in the palliative care field should consider these specific conditions in their methodology.

Therefore, this systematic review aims to summarize the current evidence, discussions and recommendations and provide methodological guidance when conducting economic evaluations in the palliative and end-of-life care setting.

OBJECTIVES

The specific objectives of this study are as follows:

For peer review only - http://bmjopen.bmj.com/site/about/guidelines.xhtml

1. To identify methodological aspects and challenges of conducting economic evaluations in the palliative and end-of-life care setting;
2. To synthesize recommendations and potential solutions to overcome these identified challenges;
3. To sum-up methodological challenges, which have remained unresolved so far; and
4. If feasible, to develop a methodological framework guideline for economic evaluations in the palliative and end-of-life-care setting.

METHODS AND ANALYSIS

Protocol

The methodology for this systematic review has been developed according to the recommendations from the Preferred Reporting Items for Systematic Review and Meta-Analysis Protocols (PRISMA-P) 2015 statement [16]. A PRISMA-P file is provided in online supplementary appendix 1.

Eligibility criteria

Population

Studies (e.g. reviews, discussion papers, and editorials) in which methodological aspects and challenges of conducting economic evaluations in the palliative and end-of-life care setting are discussed.

Comparator(s)

Economic evaluation guidelines and checklists referring to economic evaluations conducted outside the palliative and end-of-life-care setting.

Context

Palliative and end-of-life-care in different care settings such as hospitals, hospices, nursing homes, or home of the patient.

Main outcome(s)

For peer review only - http://bmjopen.bmj.com/site/about/guidelines.xhtml

The outcome of interest are methodological aspects and challenges for conducting economic evaluations in the palliative and end-of-life-care setting.

Study design

In order to serve the broad nature of our research question, we have decided a priori to develop a protocol that includes different study types published in peer-reviewed journals in this systematic review. Articles reporting on methodological aspects and challenges of conducting economic evaluations in the palliative and end-of-life-care setting are suspected being:

- Systematic reviews (and meta-analysis): a structured search has been applied and transparent and explicit methodological criteria are used to select the included studies.
- Narrative reviews: including a qualitative summary and discussion of pivotal studies known to the subject experts.
- Observational (e.g. case report or cohort), interventional studies.
- (Editorial) discussions of the literature and commentaries: including contributions to theory building or critique, arguing the case for a field of research or a course of action, or summaries of literature for the purpose of information.

Report characteristics

In this systematic review, no limit in regards to the setting will be applied, including clinical settings (e.g. hospital, nursing homes), as well as community settings or the patient's home. In case enough information can be collected for the different settings, we will present the identified information clustered per individual setting. In the field of health economics, major methodological advancements have taken place in the past 10 years, which outdate methods that were commonly applied earlier. Moreover, palliative care practice itself has undergone significant changes since 2000 [17]. For sensitivity reasons, we extended the time limit for publication one year prior to this date. All articles published after 1st January 1999 will be considered for inclusion. Articles written in English, German, French, Spanish or Dutch will be considered for inclusion.

Information sources and search strategy

A systematic search in the electronic databases MEDLINE, EMBASE, NHS Economic Evaluation Database and the Health Technology Assessment Database produced by the Centre for Reviews and

For peer review only - http://bmjopen.bmj.com/site/about/guidelines.xhtml

Dissemination will be systematically searched. Appropriate Medical Subject Headings (MeSH) terms will be selected and utilized and combined with free text words to develop a search strategy, which will then be translated to the different databases. Detailed inclusion and exclusion criteria are presented in Table 1.

Table 1 Inclusion and exclusion criteria

	Inclusion criteria	Exclusion criteria
Population	Adult palliative or end-of-life patients, irrespective of underlying disease, gender.	
Study design	Systematic reviews (and meta-analysis), narrative reviews, observational or interventional studies, (editorial) discussions and commentaries.	Economic evaluations testing the efficiency of an intervention or economic evaluations in the palliative care settings.
Outcome	Methodological aspects and recommendations for conducting economic evaluations in the palliative care setting.	
Type of publication	Articles with available full text in English, German, Spanish, French or Dutch language.	(Conference) abstracts.

For peer review only

The search syntax for MEDLINE (OVID) will be:

1 Palliative Care/
2 exp Terminal Care/
3 Terminally Ill/
4 palliat*.mp.
5 (terminal* adj6 (care or caring or ill or illness*)).ti,ab,ot,kf.
6 (end of life or last year of life or lyol or life's end).ti,ab,ot,kf.
7 advanced cancer.ti,ab,ot,kf.
8 Hospices/
9 hospice*.ti,ab,ot,kf.
10 bereave*.ti,ab,ot,kf,hw.
11 1 or 2 or 3 or 4 or 5 or 6 or 7 or 8 or 9 or 10
12 exp *Health Care Costs/
13 (health care adj3 cost*).ti,ot,kf.
14 *"Costs and Cost Analysis"/
15 Cost-Benefit Analysis/mt [Methods]
16 exp models, economic/

For peer review only - http://bmjopen.bmj.com/site/about/guidelines.xhtml

17 (economic* adj3 (evaluat* or aspect* or health or analy* or model* or framework* or frame work* or method*)).ti,ab,ot,kf,hw.
18 economics.ti,ot,kf.
19 Palliative Care/ec
20 exp Terminal Care/ec
21 Hospices/ec
22 12 or 13 or 14 or 15 or 16 or 17 or 18 or 19 or 20 or 21
23 11 and 22
24 recycl*.ti,hw,kf,jw.
25 (waste or life cycle assessment).jw.
26 24 or 25
27 23 not 26
28 limit 27 to (dutch or english or german or french or spanish)
29 limit 28 to "all child (0 to 18 years)"
30 limit 29 to "all adult (19 plus years)"
31 29 not 30
32 28 not 31
33 limit 32 to yr="1999 - 2019"

The search syntax for other databases are presented in online supplementary appendix 2.

Furthermore, we will screen the articles for any potentially relevant article they refer to in the manuscript text.

Study records

Data management

All identified references will be imported and combined in a single EndNote library. Duplicate records of the same reports will be removed.

Selection process

Two screening rounds will be conducted. In the first screening round, the title and abstract of the articles will be screened. Those articles which are selected for further inclusion based on the title and abstract screening will undergo the second screening. In the second screening round, full texts will be screened and assessed for eligibility against the pre-specified inclusion and exclusion criteria. Two reviewers will independently screen all articles, the reviewers' independent decisions on inclusion and exclusion of articles will be compared and kappa statistics calculated. Any disagreements will be discussed among the two, if the disagreement cannot be solved a third reviewer will be approached to reach a final decision. A PRISMA flow diagram [16] of the study selection process will be prepared

For peer review only - http://bmjopen.bmj.com/site/about/guidelines.xhtml

For peer review only

to present an overview of the data collection process and the decision that have been made in the course of it.

Data extraction

For the data extraction from papers standardized data extraction forms will be created (see appendix 3). One researcher will extract the data with the second researcher independently checking the accuracy of the extracted information. Relevant missing information or unclear information will be dealt with by contacting original authors of included studies. In order to ensure that the data extraction form is properly applicable, it will be pilot tested on a subsample of the total number of retrieved included studies. If questions arise, these will be discussed and if necessary, the data extraction form will be revised accordingly.

Data items

From each included article standard bibliographical information (i.e. author, journal, publication year, country) and study design will be reported. Further, information on the aspect of economic evaluation described, challenge reported, potential impact of problem described, potential solution or recommendation described, will be abstracted.

Data synthesis

A narrative synthesis of the included studies will be conducted in accordance with the Cochrane Collaboration guidelines [18]. We will critically examine the information in the included studies and categorize it according to which economic evaluation characteristic the information refers to, such as study population, study perspective, time horizon, type of economic evaluation, outcomes, data (source), costs, resource use measurement, valuation method, adjustments for inflation/deflation, discounting, ethical implications, decision modelling, care setting or other. If required, the categories will be refined or extended depending on the identified level of information and characteristic. The level of evidence for the described challenge, potential impact, concerns and solutions will be discussed per identified sub-theme. The manuscript will be prepared following the Preferred Reporting Items for Systematic Reviews and Meta-Analyses (PRISMA) guidelines.

Assessment of methodological quality

For peer review only - http://bmjopen.bmj.com/site/about/guidelines.xhtml

As the aim of this systematic review is not to determine an effect size, but to describe and synthesize a body of qualitative literature, no quality appraisal of the included studies will be conducted.

Risk of bias (quality) assessment

The efficient and correct identification of studies in which methodological aspects and challenges of conducting economic evaluations in the end-of-life care and palliative settings has an inherent risk of bias. Our research question is quiet broad, which challenges the development of a targeted search strategy, without being too narrow and missing relevant articles neither being too broad resulting in an unmanageable amount of hits. Therefore, a trained information specialist is involved in the development of the search strategy.

ETHICS AND DISSEMINATION

The systematic review will synthesize information described in published literature. Ethical approval is not required, as the proposed systematic review will not use primary data. This review will provide an overview of methodological aspects to consider when conducting an economic evaluation in the palliative and/or end-of-life-care settings. Findings will be widely disseminated through peer-reviewed publication, conference presentation(s) and via other planned dissemination activities in the course of the EU project 'the iLIVE project –live well, die well, a research programme to support living until the end' [19].

PATIENT AND PUBLIC INVOLVEMENT

This research was done without patient involvement.

DISCUSSION

This systematic review will present up-to-date findings on factors that influence the validity of economic evaluations conducted in the palliative and end-of-life care setting via summarizing and synthesizing the existing literature. With the rapid evolution of the methodology in economic evaluations, this systematic review will provide an opportunity to assess whether the recommendations for this specific field have evolved or varied over time.

For peer review only - http://bmjopen.bmj.com/site/about/guidelines.xhtml

A strength of this systematic review is its robust methodology including specific search strategy for multiple electronic databases of peer-reviewed literature, and tailored search strings carefully refined for every database searched and developed in cooperation with an information specialist. The screening process will be double screened by two individual researchers, considering articles published in English, German, Dutch, Spanish and Spanish as eligible for inclusion.

The results of this systematic review can help to clarify the current methodological questions for economic evaluations in the palliative and end-of-life care settings and may form the basis of new, setting specific methods guidelines. Findings will be relevant for researchers who which to design in the future an economic evaluation in this setting. Moreover, it can play an important role in assessing the quality of evidence from previously conducted economic evaluations focusing on palliative and end-of-life care patients.

Author affiliations

[1] Department of Health Economics, Center for Public Health, Medical University of Vienna, Kinderspitalgasse 15/1, 1090 Vienna, Austria

[2] University Library, Medical University of Vienna, Währinger Gürtel 18-20, 1090 Vienna, Austria

For peer review only

REFERENCES

1. Hofstede, J.M., et al., *Differences in palliative care quality between patients with cancer, patients with organ failure and frail patients: a study based on measurements with the consumer quality index palliative care for bereaved relatives.* Palliative medicine, 2016. **30**(8): p. 780-788.
2. De Flora, S., et al., *The epidemiological revolution of the 20th century.* The FASEB Journal, 2005. **19**(8): p. 892-897.
3. van der Velden, L.F., et al., *Dying from cancer or other chronic diseases in the Netherlands: ten-year trends derived from death certificate data.* BMC palliative care, 2009. **8**(1): p. 4.
4. Organization, W.H., *Palliative care for older people: better practices.* 2011.
5. Van den Block, L. and G. Albers, *Palliative care for older people: a public health perspective*. 2015: Oxford University Press, USA.
6. Higginson, I.J., *It would be NICE to have more evidence?* 2004, SAGE Publications Sage UK: London, England.
7. Scitovsky, A.A., *"The high cost of dying": what do the data show?* The Milbank Quarterly, 2005. **83**(4): p. 825.
8. Hogan, C., et al., *Medicare beneficiaries' costs of care in the last year of life.* Health affairs, 2001. **20**(4): p. 188-195.
9. Lubitz, J.D. and G.F. Riley, *Trends in Medicare payments in the last year of life.* New England journal of medicine, 1993. **328**(15): p. 1092-1096.
10. Hoffmann, F. and R. Rodrigues, *Informal carers: who takes care of them?* 2010.
11. Drummond, M.F., et al., *Methods for the economic evaluation of health care programmes*. 2015: Oxford university press.
12. Tuffaha, H.W., L.G. Gordon, and P.A. Scuffham, *Value of information analysis in healthcare: a review of principles and applications.* Journal of medical economics, 2014. **17**(6): p. 377-383.
13. Gomes, B., et al., *Optimal approaches to the health economics of palliative care: report of an international think tank.* Journal of pain and symptom management, 2009. **38**(1): p. 4-10.
14. Bausewein, C., et al., *Implementing patient reported outcome measures (PROMs) in palliative care-users' cry for help.* Health and quality of life outcomes, 2011. **9**(1): p. 27.
15. Higginson, I.J., et al., *Evaluating complex interventions in end of life care: the MORECare statement on good practice generated by a synthesis of transparent expert consultations and systematic reviews.* BMC medicine, 2013. **11**(1): p. 111.
16. Moher, D., et al., *Preferred reporting items for systematic review and meta-analysis protocols (PRISMA-P) 2015 statement.* Systematic reviews, 2015. **4**(1): p. 1.
17. Dy, S.M., et al., *Closing the quality gap: revisiting the state of the science (vol. 8: improving health care and palliative care for advanced and serious illness).* Evidence report/technology assessment, 2012(2088): p. 1.
18. Ryan, R., *Cochrane Consumers and Communication Review Group: data synthesis and analysis.* Cochrane Consumers and Communication Review Group, available at: http://cccrg. cochrane. org [Accessed 8 March 2016].[Google Scholar], 2013.
19. *The iLIVE project live well-die well, a research programme to support living until the end* 2019; Available from: https://www.iliveproject.eu/the-consortium [Accessed 30.09.2019].

For peer review only - http://bmjopen.bmj.com/site/about/guidelines.xhtml

AUTHORS’ CONTRIBUTIONS

JS and CF conceived of the idea, developed the research question, study methods and data extraction form. EC and CF designed and developed the search strategy and strings. CF drafted the manuscript with contribution from JS. All authors approved the final manuscript.

FUNDING STATEMENT

This project has received funding from the European Union´s Horizon 2020 research and innovation programme under grant agreement No 825731.

PATIENT CONSENT

Not required.

COMPETING INTERESTS STATEMENT

The authors declare that they have no conflict of interest.

For peer review only

For peer review only

For peer review only - http://bmjopen.bmj.com/site/about/guidelines.xhtml

APPENDIX

Appendix 1

PRISMA-P (Preferred Reporting Items for Systematic review and Meta-Analysis Protocols) 2015 checklist: recommended items to address in a systematic review protocol*

Section and topic	Item No	Checklist item	Reported
**ADMINISTRATIVE INFORMATION**			
Title:			
Identification	1a	Identify the report as a protocol of a systematic review	Title page
Update	1b	If the protocol is for an update of a previous systematic review, identify as such	N/A
Registration	2	If registered, provide the name of the registry (such as PROSPERO) and registration number	Page 2
Authors:			
Contact	3a	Provide name, institutional affiliation, e-mail address of all protocol authors; provide physical mailing address of corresponding author	Title page
Contributions	3b	Describe contributions of protocol authors and identify the guarantor of the review	Page 10
Amendments	4	If the protocol represents an amendment of a previously completed or published protocol, identify as such and list changes; otherwise, state plan for documenting important protocol amendments	N/A
Support:			
Sources	5a	Indicate sources of financial or other support for the review	Page 10
Sponsor	5b	Provide name for the review funder and/or sponsor	Page 10
Role of sponsor or funder	5c	Describe roles of funder(s), sponsor(s), and/or institution(s), if any, in developing the protocol	Page 10
**INTRODUCTION**			
Rationale	6	Describe the rationale for the review in the context of what is already known	Page 3
Objectives	7	Provide an explicit statement of the question(s) the review will address with reference to participants, interventions, comparators, and outcomes (PICO)	Page 3-4
**METHODS**			
Eligibility criteria	8	Specify the study characteristics (such as PICO, study design, setting, time frame) and report characteristics (such as years considered, language, publication status) to be used as criteria for eligibility for the review	Page 4-6

For peer review only

Information sources	9	Describe all intended information sources (such as electronic databases, contact with study authors, trial registers or other grey literature sources) with planned dates of coverage	Page 5
Search strategy	10	Present draft of search strategy to be used for at least one electronic database, including planned limits, such that it could be repeated	Page 6-7 Appendix 2
Study records:			
Data management	11a	Describe the mechanism(s) that will be used to manage records and data throughout the review	Page 6
Selection process	11b	State the process that will be used for selecting studies (such as two independent reviewers) through each phase of the review (that is, screening, eligibility and inclusion in meta-analysis)	Page 7-8
Data collection process	11c	Describe planned method of extracting data from reports (such as piloting forms, done independently, in duplicate), any processes for obtaining and confirming data from investigators	Page 8
Data items	12	List and define all variables for which data will be sought (such as PICO items, funding sources), any pre-planned data assumptions and simplifications	Page 4-6
Outcomes and prioritization	13	List and define all outcomes for which data will be sought, including prioritization of main and additional outcomes, with rationale	Page 4 and 6
Risk of bias in individual studies	14	Describe anticipated methods for assessing risk of bias of individual studies, including whether this will be done at the outcome or study level, or both; state how this information will be used in data synthesis	Page 9
Data synthesis	15a	Describe criteria under which study data will be quantitatively synthesised	Page 8
	15b	If data are appropriate for quantitative synthesis, describe planned summary measures, methods of handling data and methods of combining data from studies, including any planned exploration of consistency (such as I^2, Kendall's τ)	N/A
	15c	Describe any proposed additional analyses (such as sensitivity or subgroup analyses, meta-regression)	N/A
	15d	If quantitative synthesis is not appropriate, describe the type of summary planned	N/A
Meta-bias(es)	16	Specify any planned assessment of meta-bias(es) (such as publication bias across studies, selective reporting within studies)	N/A
Confidence in cumulative evidence	17	Describe how the strength of the body of evidence will be assessed (such as GRADE)	Page 9

* It is strongly recommended that this checklist be read in conjunction with the PRISMA-P Explanation and Elaboration (cite when available) for important clarification on the items. Amendments to a review protocol should be tracked and dated. The copyright for PRISMA-P (including checklist) is held by the PRISMA-P Group and is distributed under a Creative Commons Attribution Licence 4.0.

For peer review only

Appendix 2

Search syntax in different databases

EMBASE.com

#35 #28 NOT #33 AND [1999-2019]/py

#34 #28 NOT #33

#33 #29 NOT #32

#32 #30 OR #31

#31 adult*:ti OR elderly:ti OR 'old* people':ti

#30 (child*:ti OR infan*:ti OR newborn*:ti OR neonat*:ti OR juvenile*:ti OR adolescen*:ti OR youth*:ti OR pediat*:ti OR paediat*:ti) AND ([young adult]/lim OR [adult]/lim OR [middle aged]/lim OR [aged]/lim OR [very elderly]/lim)

#29 child*:ti OR infan*:ti OR newborn*:ti OR neonat*:ti OR juvenile*:ti OR adolescen*:ti OR youth*:ti OR pediat*:ti OR paediat*:ti

#28 #27 AND ([dutch]/lim OR [english]/lim OR [german]/lim OR [spanish]/lim OR [french]/lim)

#27 #23 NOT #26

#26 #24 OR #25

#25 waste:jt OR 'life cycle assessment':jt

#24 recycl*:ti,de,kw,jt

#23 #13 AND #22

#22 #14 OR #15 OR #16 OR #17 OR #18 OR #19 OR #20 OR #21

#21 ('health care' NEAR/3 cost*):ti,kw

#20 economics:ti,kw

(economic* NEAR/3 (evaluat* OR aspect* OR health OR analy* OR model* OR framework* OR

#19 'frame work*' OR method*)):ti,ab,kw

#18 'health care cost'/exp/mj

#17 'economics'/mj

For peer review only

#16 'economic model'/de

#15 'health economics'/de

#14 'economic evaluation'/de

#13 #1 OR #2 OR #3 OR #4 OR #5 OR #6 OR #7 OR #8 OR #9 OR #10 OR #11 OR #12

#12 bereave*:de,ti,ab,kw

#11 hospice*:de,ti,ab,kw

#10 'advanced cancer':ti,ab,kw

#9 'end of life':de,ti,ab,kw OR 'last year of life':de,ti,ab,kw OR 'lyol':de,ti,ab,kw OR 'life* end':de,ti,ab,kw

#8 (terminal* NEAR/6 (care* OR caring OR ill OR illness* OR patient*)):ti,ab,kw

#7 palliat*:ti,ab,kw

#6 'hospice'/de

#5 'terminal disease'/de

#4 'palliative therapy'/exp

#3 'terminal care'/exp

#2 'terminally ill patient'/exp

#1 'palliative nursing'/de

For peer review only

EBM Reviews - Health Technology Assessment Database (OVID) (discontinued at the end of 2016)

1 Palliative Care/

2 exp Terminal Care/

3 terminally ill/

4 palliat*.mp.

5 (terminal* adj6 (care or caring or ill or illness*)).mp.

6 (end of life or last year of life or lyol or life* end).mp.

7 advanced cancer.mp.

8 hospices/

9 hospice*.mp.

10 bereave*.mp.

11 1 or 2 or 3 or 4 or 5 or 6 or 7 or 8 or 9 or 10

12 exp Health Care Costs/

13 (health care adj3 cost*).mp.

14 "Costs and Cost Analysis"/

15 Cost-Benefit Analysis/mt [Methods]

16 exp models, economic/

17 (economic* adj3 (evaluat* or aspect* or health or analy* or model* or framework* or frame work* or method*)).mp.

18 economics.mp.

19 12 or 13 or 14 or 15 or 16 or 17 or 18

20 11 and 19

21 limit 20 to "all child (0 to 18 years)"

22 limit 20 to (dutch or english or german or spanish or french)

EBM Reviews - NHS Economic Evaluation Database (OVID) (discontinued at the 31st March 2015)

1 Palliative Care/

2 exp Terminal Care/

3 terminally ill/

4 palliat*.mp. [mp=title, text, subject heading word]

5 (terminal* adj6 (care or caring or ill or illness*)).mp. [mp=title, text, subject heading word]

6 (end of life or last year of life or lyol or life* end).mp. [mp=title, text, subject heading word]

7 advanced cancer.mp. [mp=title, text, subject heading word]

8 hospices/

9 hospice*.mp. [mp=title, text, subject heading word]

For peer review only

10	bereave*.mp. [mp=title, text, subject heading word]
11	1 or 2 or 3 or 4 or 5 or 6 or 7 or 8 or 9 or 10
12	limit 11 to "all child (0 to 18 years)"
13	limit 12 to "all adult (19 plus years)"
14	12 not 13
15	11 not 14
16	limit 15 to yr="1999 - 2015"

For peer review only

Appendix 3

Data extraction form

**Reviewer:** ………………………………		**Date:** ………………………………	
**Author:** ………………………………		**Country:** ………………………………	
**Journal:** ………………………………		**Manuscript code:** ………………………………	
**Date of publication:** ………………………………		**Funding source:** ………………………………	
**Study design:**	O Case report	O Cohort	O Review (scoping, systematic, narrative)
	O Editorial/ commentary / letter	O Other: ……………………………………………………………….	
**Aspect of economic evaluation (EE) described:**	O Study population	O Study perspective	O Time horizon
**O Type of EE**	O Outcomes	O Data (source)	O Costs
**O Resource use measurement**	O Valuation method	O Adjustments for inflation/deflation	O Discounting
**O Ethical implications**	O Decision modelling	O Care setting	O Other: ……………
**Methodological problem described:** ………………………………………………………………………………………………			
**Potential impact of problem described on results of EE:** ………………………………………………………………………………………………			
**Potential solution suggested in study:** ………………………………………………………………………………………………			

For peer review only

PRISMA-P (Preferred Reporting Items for Systematic review and Meta-Analysis Protocols) 2015 checklist: recommended items to address in a systematic review protocol*

Section and topic	Item No	Checklist item	Reported
**ADMINISTRATIVE INFORMATION**			
Title:			
Identification	1a	Identify the report as a protocol of a systematic review	Title page
Update	1b	If the protocol is for an update of a previous systematic review, identify as such	N/A
Registration	2	If registered, provide the name of the registry (such as PROSPERO) and registration number	Page 2
Authors:			
Contact	3a	Provide name, institutional affiliation, e-mail address of all protocol authors; provide physical mailing address of corresponding author	Title page
Contributions	3b	Describe contributions of protocol authors and identify the guarantor of the review	Page 10
Amendments	4	If the protocol represents an amendment of a previously completed or published protocol, identify as such and list changes; otherwise, state plan for documenting important protocol amendments	N/A
Support:			
Sources	5a	Indicate sources of financial or other support for the review	Page 10
Sponsor	5b	Provide name for the review funder and/or sponsor	Page 10
Role of sponsor or funder	5c	Describe roles of funder(s), sponsor(s), and/or institution(s), if any, in developing the protocol	Page 10
**INTRODUCTION**			
Rationale	6	Describe the rationale for the review in the context of what is already known	Page 3
Objectives	7	Provide an explicit statement of the question(s) the review will address with reference to participants, interventions, comparators, and outcomes (PICO)	Page 3-4
**METHODS**			
Eligibility criteria	8	Specify the study characteristics (such as PICO, study design, setting, time frame) and report characteristics (such as years considered, language, publication status) to be used as criteria for eligibility for the review	Page 4-6
Information sources	9	Describe all intended information sources (such as electronic databases, contact with study authors, trial registers or other grey literature sources) with planned dates of coverage	Page 5
Search strategy	10	Present draft of search strategy to be used for at least one electronic database, including planned limits, such that it could be repeated	Page 6-7 Appendix 2

For peer review only

Study records:			
Data management	11a	Describe the mechanism(s) that will be used to manage records and data throughout the review	Page 6
Selection process	11b	State the process that will be used for selecting studies (such as two independent reviewers) through each phase of the review (that is, screening, eligibility and inclusion in meta-analysis)	Page 7-8
Data collection process	11c	Describe planned method of extracting data from reports (such as piloting forms, done independently, in duplicate), any processes for obtaining and confirming data from investigators	Page 8
Data items	12	List and define all variables for which data will be sought (such as PICO items, funding sources), any pre-planned data assumptions and simplifications	Page 4-6
Outcomes and prioritization	13	List and define all outcomes for which data will be sought, including prioritization of main and additional outcomes, with rationale	Page 4 and 6
Risk of bias in individual studies	14	Describe anticipated methods for assessing risk of bias of individual studies, including whether this will be done at the outcome or study level, or both; state how this information will be used in data synthesis	Page 9
Data synthesis	15a	Describe criteria under which study data will be quantitatively synthesised	Page 8
	15b	If data are appropriate for quantitative synthesis, describe planned summary measures, methods of handling data and methods of combining data from studies, including any planned exploration of consistency (such as I^2, Kendall's τ)	N/A
	15c	Describe any proposed additional analyses (such as sensitivity or subgroup analyses, meta-regression)	N/A
	15d	If quantitative synthesis is not appropriate, describe the type of summary planned	N/A
Meta-bias(es)	16	Specify any planned assessment of meta-bias(es) (such as publication bias across studies, selective reporting within studies)	N/A
Confidence in cumulative evidence	17	Describe how the strength of the body of evidence will be assessed (such as GRADE)	Page 9

* It is strongly recommended that this checklist be read in conjunction with the PRISMA-P Explanation and Elaboration (cite when available) for important clarification on the items. Amendments to a review protocol should be tracked and dated. The copyright for PRISMA-P (including checklist) is held by the PRISMA-P Group and is distributed under a Creative Commons Attribution Licence 4.0.

For peer review only

BMJ Open

Methodologic aspects of economic evaluations conducted in the palliative or end of life care settings: a systematic review protocol

Journal:	*BMJ Open*
Manuscript ID	bmjopen-2019-035760.R1
Article Type:	Protocol
Date Submitted by the Author:	17-Feb-2020

Complete List of Authors:	Fischer, Claudia; Medical University of Vienna Center for Public Health, Health Economics<br>Chwala, Eva; Medical University of Vienna, University Library<br>Simon, Judit; Medical University of Vienna Center for Public Health, Health Economics

**Primary Subject Heading**:	Palliative care
Secondary Subject Heading:	Palliative care, Research methods, Health economics
Keywords:	PALLIATIVE CARE, STATISTICS & RESEARCH METHODS, HEALTH ECONOMICS, Adult palliative care < PALLIATIVE CARE

SCHOLARONE™
Manuscripts

I, the Submitting Author has the right to grant and does grant on behalf of all authors of the Work (as defined in the below author licence), an exclusive licence and/or a non-exclusive licence for contributions from authors who are: i) UK Crown employees; ii) where BMJ has agreed a CC-BY licence shall apply, and/or iii) in accordance with the terms applicable for US Federal Government officers or employees acting as part of their official duties; on a worldwide, perpetual, irrevocable, royalty-free basis to BMJ Publishing Group Ltd ("BMJ") its licensees and where the relevant Journal is co-owned by BMJ to the co-owners of the Journal, to publish the Work in this journal and any other BMJ products and to exploit all rights, as set out in our licence.

The Submitting Author accepts and understands that any supply made under these terms is made by BMJ to the Submitting Author unless you are acting as an employee on behalf of your employer or a postgraduate student of an affiliated institution which is paying any applicable article publishing charge ("APC") for Open Access articles. Where the Submitting Author wishes to make the Work available on an Open Access basis (and intends to pay the relevant APC), the terms of reuse of such Open Access shall be governed by a Creative Commons licence – details of these licences and which Creative Commons licence will apply to this Work are set out in our licence referred to above.

Other than as permitted in any relevant BMJ Author's Self Archiving Policies, I confirm this Work has not been accepted for publication elsewhere, is not being considered for publication elsewhere and does not duplicate material already published. I confirm all authors consent to publication of this Work and authorise the granting of this licence.

For peer review only

Methodologic aspects of economic evaluations conducted in the palliative or end of life care settings: a systematic review protocol

Claudia Fischer[1], Eva Chwala[2], Judit Simon[1]

Corresponding author: Claudia Fischer[1]

E-mail address of corresponding author: Claudia.fischer@meduniwien.ac.at

For peer review only

For peer review only - http://bmjopen.bmj.com/site/about/guidelines.xhtml

ABSTRACT

Introduction: In light of this growing palliative care and end of life care patient population, as well as new (expensive) drugs and treatments, quality research providing evidence for decision-making is required. However, common research guidance is lacking in this field, especially in respect to the methods applied in economic evaluations. Therefore, the aim of the planned systematic review is to identify and summarize relevant information on methodological challenges, potential solutions and recommendations for conducting economic evaluations of interventions in adult patients, irrespective of their underlying disease and gender in the palliative or end of life care settings, with no restrictions in regards to countries/geographic regions. The results of this systematic review may help to clarify the current methodological questions and form the basis of new, setting specific methods guidelines and support ongoing applied economic evaluations in the field.

Methods and analysis: A systematic review will be conducted using MEDLINE, EMBASE, Health Technology Assessment Database and NHS Economic Evaluation Database to identify studies published from 1999 onwards with relevant information on methodological challenges, potential solutions and recommendations for conducting economic evaluations in the palliative or end of life care settings. Articles in English, German, Spanish, French or Dutch language will be considered. Two independent reviewers will conduct the screening of articles; any discrepancies will be resolved by discussion and involvement of a third reviewer. Pre-designed data extraction forms will be applied, consequently narratively synthesized and categorized. Studies methodological quality will be critically appraised In addition, also existing economic guidelines and checklists for specific information on the palliative and end of life care sector will be searched.

Ethics and dissemination: Ethical approval is not required, as this is a planned systematic review of published literature. An article will be disseminated in a related peer-reviewed journal, as well as presented at leading palliative care and health economic conferences.

Acknowledgements: The `iLIVE project – live well, die well, a research programme to support living until the end´ has received funding from the European Union’s Horizon 2020 research and innovation programme under grant agreement No 825731.

STRENGTHS AND LIMITATIONS OF THIS STUDY

- To our knowledge, this systematic review, which will be conducted and reported according to the current highest methodological standard, is the first one to identify methodological

For peer review only - http://bmjopen.bmj.com/site/about/guidelines.xhtml

aspects and challenges to consider when conducting an economic evaluation in the palliative or end of life care settings.

- Our research question is quiet broad with numerous challenges in the development of a targeted search strategy without this being too narrow or too broad. Therefore, a robust methodology including specific search strategy for multiple electronic databases of peer-reviewed literature, and tailored search strings carefully refined for every database searched and developed in cooperation with an information specialist have been developed and piloted.
- Further, the screening process will be carried out by three individual researchers, considering articles published in multiple languages (English, German, French, Dutch, and Spanish) for inclusion. While this language restriction might present a potential limitation, this shall be only marginal in the high income setting considering also the good coverage of countries with strong track record in economic evaluations and palliative/end of life care aspects. A separate review tailored to low and middle-income settings may become necessary if the current review identifies relevant cultural/spiritual/preference aspects important also for economic evaluations. Formal transferability assessment will be carried out as far as possible.
- This systematic review will be carried out alongside a large EU project (the iLIVE project), which allows direct testing of our findings and develop practice-applied recommendations for this area. Further, the iLIVE project includes different relevant stakeholders, which enables a comprehensive external review of our findings. Specifically, we will ensure to involve the iLIVE PP advisory board representatives in the interpretation of the review findings.

For peer review only

INTRODUCTION

Over the past century, patterns of mortality in the Western world have changed [1]. While sudden death, mainly due to infectious disease, used to be the major cause of death, nowadays there is an increase of the ageing population living with advanced stages of incurable chronic conditions such as cancer, chronic heart failure or chronic obstructive pulmonary disease (COPD) near the end of life [2, 3].

It is crucial to enable this growing patient group to live well at the end of their lives as well as to die well [4, 5]. This requires quality research providing evidence for decisions on clinical guidelines,

For peer review only - http://bmjopen.bmj.com/site/about/guidelines.xhtml

treatments and services [6]. In light of the new (expensive) drugs and interventions, an effective and efficient resource allocation is of great importance in the palliative and end of life care setting [7]. Studies have estimated the cost of care in the last year of life at 25 to 30% of all medical expenditures during a lifetime [8, 9]. This number is thought to even further increase in light of the ageing population and the prospect of potentially reduced number of informal carers in the future [10].

Economic evaluations are the comparative analysis of alternative courses of action in terms of both, their costs and consequences [11], and have been widely applied in other fields to answer questions in regards to cost-effectiveness of health care interventions and inform decision-makers and commissioners [12]. Nevertheless, the economic component has often been overlooked in palliative care or end of life care studies, leaving a number of unanswered questions for the quality assurance, monitoring, funding, and evaluation of palliative care or end of life care [13, 14]. Palliative and end of life care research lack common research guidance [15], which would be essential as these settings differ from other health care fields in several aspects, which is why existing methods of economic evaluations may not be suitable for them. For example, compared to interventions in other fields, the ultimate aim of interventions in the palliative or end of life care settings is not to improve quality of life and the extension of it but rather focus on the quality of dying, patient's dignity or strengthening relationships with loved ones [16, 17]. Therefore, the appropriateness of generic quality of life (QALY) measures for economic evaluations in the palliative or end of life care settings may be questioned, as their measured quality dimensions may not reflect those dimensions relevant for palliative or end of life care patients [18].

Further, it is well known that informal carers play an important role in the care of patients in many disease fields, such as chronic conditions [19]. The contribution of informal carers in the care of patients in the palliative or end of life care fields is thought to be substantially high [20, 21]. Although the general acknowledgement of their importance, often their contributions, both in respect to costs and outcomes, are not included in economic evaluations [22, 23]. This is due to diverse reasons, for instance, 1) payers want a perspective applied that reflects costs that actually emerge for them [24], 2) there is no gold standard how to value (i.e. attribute a monetary value to) indirect costs of informal care [25-27], 3) ethical concerns about involving participants in data assessment and research at a suitable time, especially in regards to finances, which may add to the already enormous emotional and physical burden they face [28]. However, leaving these costs and outcomes unconsidered, may result in a biased reflection of the true costs and benefits of interventions in the palliative or end of life care settings [29].

For peer review only - http://bmjopen.bmj.com/site/about/guidelines.xhtml

In order to generate valid und useful evidence and decision base, the research methods of economic evaluations applied in the palliative care or end of life care fields should consider these specific conditions in their methodology. Therefore, this systematic review aims to summarize the methodological aspects and challenges (e.g. valuation of informal care, usability of QALYs or other outcome types in this field) and recommendations as well as potential solutions to overcome these problems and consequently provide methodological guidance when conducting economic evaluations in the palliative or end of life care settings, irrespective of the underlying disease, gender or geographic region.

OBJECTIVES

The specific objectives of this study are as follows:

1. To identify methodological aspects and challenges of conducting economic evaluations in palliative or end of life care patients irrespective of their underlying disease, gender nor their geographic region;
1. To synthesize recommendations and potential solutions to overcome these identified challenges;
2. To sum-up methodological challenges, which have remained unresolved so far; and
3. If feasible, to develop a methodological framework guideline for economic evaluations in the palliative and end of life care settings;

METHODS AND ANALYSIS

Protocol

This systematic review protocol has been developed according to the recommendations from the Preferred Reporting Items for Systematic Review and Meta-Analysis Protocols (PRISMA-P) 2015 statement [30] and PRISMA for Abstracts checklist [31]. A PRISMA-P file and PRISMA for Abstracts checklist is provided in online supplementary appendix 1. In addition, the review will follow the 5-step approach on the state-of-the-art methodology for conducting systematic reviews of economic evidence by Van Mastrigt et al. (2016) [32].

For peer review only - http://bmjopen.bmj.com/site/about/guidelines.xhtml

The work on the described systematic review has been started in June 2019 (conceptualization of the idea of the systematic review) and will take approximately until June 2021 (finalization of the data synthesis and write up of results).

Eligibility criteria

Population

Adult (a person older than 19 years of age) palliative or end of life patients, irrespective of underlying disease, gender.

Comparator(s)

Not specified.

Context

Palliative and end of life care in different care settings such as hospitals, hospices, nursing homes, or home of the patient. No explicit restrictions in regards to countries/geographic regions are made. However, due to some necessary language restrictions, it is likely that generalisability of the overall findings will be limited to Europe/North America/South America and Australia. Context specificity and transferability will be further assessed based on the findings.

Main outcome(s)

The outcome of interest are methodological aspects and challenges (e.g. valuation of informal care, usability of QALYs or other outcome types in this field) for conducting economic evaluations in the palliative or end of life care settings.

Study design

In order to serve the broad nature of our research question, we have decided a priori to develop a protocol that includes different study types published in peer-reviewed journals in this systematic review. Articles reporting on methodological aspects and challenges of conducting economic evaluations in the palliative or end of life care setting are suspected being:

For peer review only

For peer review only - http://bmjopen.bmj.com/site/about/guidelines.xhtml

- Systematic reviews (and meta-analysis): a structured search has been applied and transparent and explicit methodological criteria are used to select the included studies.
- Narrative reviews: including a qualitative summary and discussion of pivotal studies known to the subject experts.
- Observational (e.g. case report or cohort), interventional studies.
- (Full) economic evaluations.
- Economic guidelines and checklists.
- (Editorial) discussions of the literature and commentaries: including contributions to theory building or critique, arguing the case for a field of research or a course of action, or summaries of literature for the purpose of information.
- Qualitative studies.

Report characteristics

In this systematic review, no limit in regards to the setting will be applied, including clinical settings (e.g. hospital, nursing homes), as well as community settings or the patient's home. Depending on whether we will be able to identify sufficient information per individual setting in which care may be provided, we will present the identified information clustered per individual care setting, otherwise no distinction per setting will be undertaken. In the field of health economics, major methodological advancements have taken place in the past 10 years, which outdate methods that were commonly applied earlier. Moreover, palliative care practice itself has undergone significant changes since 2000 [33]. For sensitivity reasons, we extended the time limit for publication one year prior to this date. All articles published after 1st January 1999 will be considered for inclusion. Articles written in English, German, French, Spanish or Dutch will be considered for inclusion.

The authors are able to read and analyze articles in English, German, and Dutch. Further, the iLIVE project consortium includes native speakers who will provide help with articles of interest in Spanish and French.

Information sources and search strategy

In collaboration with an information specialist, a systematic search in the electronic databases MEDLINE, EMBASE, NHS Economic Evaluation Database and the Health Technology Assessment Database produced by the Centre for Reviews and Dissemination will be systematically conducted. Appropriate Medical Subject Headings (MeSH) terms will be selected and utilized and combined with free text words to develop a search strategy, which will then be translated to the different databases. The developed search strategy will be based on a review of published search filters for palliative care

For peer review only - http://bmjopen.bmj.com/site/about/guidelines.xhtml

and health economics (CADTH economic search filter https://www.cadth.ca/resources/finding-evidence/strings-attached-cadths-database-search-filters#eco, OVID Expert Searches Health Science http://resourcecenter.ovid.com/site/resources/expert_search/healthexp.html, CareSearch Palliative Care search filter https://www.caresearch.com.au/caresearch/tabid/377/Default.aspx, PubMed Health Sciences Research Queries https://www.nlm.nih.gov/nichsr/hedges/HSR_queries_table.html, SIGN Search filter https://www.sign.ac.uk/search-filters.html, McMaster Search filter https://hiru.mcmaster.ca/hiru/HIRU_Hedges_MEDLINE_Strategies.aspx) [34-38]. Different combinations of search terms and specifications have been tested in regards to their sensitivity for our research question. For each applied modification, the first 100 articles have been evaluated to decide whether the modification is applied or not.

Detailed inclusion and exclusion criteria are presented in Table 1.

Table 1 Inclusion and exclusion criteria

	Inclusion criteria	Exclusion criteria
Population	Adult* palliative or end of life patients, irrespective of underlying disease, gender.	
Study design	Systematic reviews (and meta-analysis), narrative reviews, observational or interventional studies, (editorial) discussions and commentaries, (full) economic evaluations, qualitative studies.	~~Economic evaluations testing the efficiency of an intervention or economic evaluations in the palliative care settings.~~
Outcome	Methodological aspects and recommendations for conducting economic evaluations in the palliative and end of life care settings.	
Type of publication	Articles with available full text in English, German, Spanish, French or Dutch language.	(Conference) abstracts.

*Following the WHO definition, an adult is a person older than 19 years of age [39]

For peer review only - http://bmjopen.bmj.com/site/about/guidelines.xhtml

The final search syntax for MEDLINE (OVID) is a modification of the reviewed search strings of Gomes et al. (2016)[38] and Rietjens et al. (2019)[34]:

1 Palliative Care/
2 exp Terminal Care/
3 Terminally Ill/
4 palliat*.mp.
5 (terminal* adj6 (care or caring or ill or illness*)).ti,ab,ot,kf.
6 (end of life or last year of life or lyol or life's end).ti,ab,ot,kf.
7 advanced cancer.ti,ab,ot,kf.
8 Hospices/
9 hospice*.ti,ab,ot,kf.
10 bereave*.ti,ab,ot,kf,hw.
11 1 or 2 or 3 or 4 or 5 or 6 or 7 or 8 or 9 or 10
12 exp *Health Care Costs/
13 ((health care or healthcare) adj3 cost*).ti,ot,kf,kw.
14 *"Costs and Cost Analysis"/
15 Cost-Benefit Analysis/mt [Methods]
16 exp models, economic/
17 (economic* adj3 (evaluat* or aspect* or health or analy* or model* or framework* or frame work* or method*)).ti,ab,ot,kf,hw.
18 economics.ti,ot,kf.
19 Palliative Care/ec
20 exp Terminal Care/ec
21 Hospices/ec
22 12 or 13 or 14 or 15 or 16 or 17 or 18 or 19 or 20 or 21
23 11 and 22
24 recycl*.ti,hw,kf,jw.
25 (waste or life cycle assessment).jw.
26 24 or 25
27 23 not 26
28 limit 27 to (dutch or english or german or french or spanish)
29 limit 28 to "all child (0 to 18 years)"
30 limit 29 to "all adult (19 plus years)"
31 29 not 30
32 28 not 31
33 limit 32 to yr="1999 - 2019"

The search syntax for other databases are presented in online supplementary appendix 2.

In addition, we will screen relevant websites (standard health economic evaluation associations (e.g. ISPOR, iHEA), the resources of the members area of the International Collaborative for Best Care for the Dying Person, as well as reports of HTA bodies (e.g. NICE) to identify potential relevant guidelines to conduct economic evaluations in the palliative or end of life care settings, as well as specific checklist for quality appraisal and value frameworks.

For peer review only

Furthermore, we will screen the articles for any potentially relevant article they refer to in the manuscript text.

Study records

Data management

For transparency and reproducibility a clear documentation of all searches, the electronic databases searches, the references searched, and the hand search gray literature, will be kept. The search syntaxes translated for all searched databases will be made available in the appendix of the systematic review, a PRISMA flow chart will be presented, illustrating the numbers of records retrieved and selection flow through the screening rounds. All identified references will be imported and combined in a single EndNote library. Duplicate records of the same reports will be removed.

Selection process

Two screening rounds will be conducted. In the first screening round, the title and abstract of the articles will be screened. Two reviewers will independently screen all articles, the reviewers' independent decisions on inclusion and exclusion of articles will be compared and kappa statistics calculated. In case of the absence of an article's title/abstract, the full text of the article will be directly screened for potential inclusion. Those articles which are selected for further inclusion based on the title and abstract screening will undergo the second screening. In the second screening round, full texts will be screened and assessed for eligibility against the pre-specified inclusion and exclusion criteria. Any disagreements will be discussed among the two, if the disagreement cannot be solved a third reviewer will be approached to reach a final decision. A PRISMA flow diagram [30] of the study selection process will be prepared to present an overview of the data collection process and the decision that have been made in the course of it.

Data extraction

For the data extraction from papers standardized data extraction forms will be created in Excel (see appendix 3 for illustration of categories). One researcher will extract the data with the second researcher independently checking the accuracy of the extracted information. Relevant missing information or unclear information will be dealt with by contacting original authors of included studies. In order to ensure that the data extraction form is properly applicable, it will undergo

For peer review only - http://bmjopen.bmj.com/site/about/guidelines.xhtml

piloting, testing it for its user-friendliness and completeness on a subsample of the total number of retrieved included studies. If questions arise, these will be discussed and if necessary, the data extraction form will be revised accordingly.

Data items

From each included article describing methodological aspects of economic evaluations in the palliative and end of life care settings standard bibliographical information (i.e. author, journal, publication year, country) and study design will be reported. Further, information on the aspect of economic evaluation described, challenge reported, potential impact of problem described, potential solution or recommendation described, will be abstracted. We will also collect information (e.g. study characteristics and patient characteristics) from the identified applied economic evaluation. The assessed outcomes and cost categories considered in the economic evaluation will be abstracted and summarized.

Data synthesis

A narrative synthesis of the included studies will be conducted in accordance with the Cochrane Collaboration guidelines [40] for which two data extraction sheets in Excel will be prepared, one for studies on methodological studies and one for applied economic evaluations. For the data extraction sheet for methodological studies, we a priori defined major economic evaluation characteristics by using the Consolidated Health Economic Evaluation Reporting Standards (Cheers) Checklist as a base [41]. For methodological/qualitative papers, such lists do not exist. Instead, we pre-defined a set of attributes in our data extraction sheet that we will collect as standard. Of course, these categories will remain open for modification and extension, depending on which information we will identify in the data extraction and analysis phase. If required, the categories will be refined or extended depending on the identified level of information and characteristic. The level of evidence for the described challenge, potential impact, concerns and solutions will be discussed per identified sub-theme. With the help of the second data extraction sheet, which has been developed specifically for applied economic evaluations, the characteristics of the identified economic evaluations will be summarized as well as overviews of the considered outcomes and cost categories created.

For included articles, which have been executed in which the specific country and hence the specific health care system is of relevance, the transferability of study findings will be assessed using transferability checklists [42].

For peer review only - http://bmjopen.bmj.com/site/about/guidelines.xhtml

In case we are able to find sufficient information in regards to recommendations and potential solutions in respect to the challenges identified, we will develop a methodological framework guideline for economic evaluations in the palliative or end of life care setting in form of a critical appraisal checklist of good practice, aiming to improve the design, the collection of data (i.e. outcomes, and costs) as well as analysis of data in economic evaluations in this field. The methodological framework will emphasize on a set of recommendations, which differ from those when conducting economic evaluation in settings outside the palliative or end of life care settings.

The manuscript will be prepared following the Preferred Reporting Items for Systematic Reviews and Meta-Analyses (PRISMA) guidelines.

Assessment of methodological quality and risk of bias

In order to be able to appraise the overall quality of an evidence, base, and the strength of conclusion drawn from it, studies methodological quality will be critically appraised. Formal quality checks will be carried out where relevant lists are readily available (e.g. NICE Quality appraisal checklists [43], CHEERS checklist [41] or PRISMA checklist [44]) and will record the quality/risk of bias information in our data extraction sheet. Two reviewers will be involved in the risk of bias assessment, one will fill in the quality appraisals, the second one will conduct quality checks, discrepancies will be solved by discussions and if necessary, consultation of a third reviewer. Once the extraction is finalized, we will decide on how this information can formally be incorporated into the synthesis of the evidence once data extraction is finalized and we have a clearer overview of the feasibility.

ETHICS AND DISSEMINATION

The systematic review will synthesize information described in published literature. Ethical approval is not required, as the proposed systematic review will not use primary data. This review will provide an overview of methodological aspects to consider when conducting an economic evaluation in the palliative and end of life care settings. Findings will be widely disseminated through peer-reviewed publication, conference presentation(s) and via other planned dissemination activities in the course of the EU project ‘the iLIVE project –live well, die well, a research programme to support living until the end’ [45].

For peer review only - http://bmjopen.bmj.com/site/about/guidelines.xhtml

PATIENT AND PUBLIC INVOLVEMENT

Our results and the developed framework will be undergo a review process, i.e. discussed and potentially refined with the experts within the iLIVE consortium and subsequently also with the palliative and end of life care community at international scientific conference discussions. Specifically, we will ensure to involve the iLIVE PP advisory board representatives in the interpretation of the review findings. As recommended by Van Mastrigt et al (2017) these reviewers will not have been involved in the development of the framework and will include experts in the clinical area, methodological or HTA expert, or the targeted patient population [32].

DISCUSSION

To our knowledge, this will be the first systematic review to identify methodological aspects and challenges to consider when conducting an economic evaluation in the palliative or end of life care settings.

The systematic overview of the identified methodological aspects and challenges of conducting economic evaluations in the palliative and end of life care settings will help to deepen understanding of the challenge to conduct economic evaluations in these settings. The aim is to provide a systematic and comprehensive overview of the current existing evidence base. For instance, provide an overview of the currently used outcome measures used in economic evaluations in the palliative and end of life care settings, provide a summary of their advantages and disadvantages, and the discussion whether or not generic measures may be used in these settings and provide an overview of available evaluation studies. The overview should help authors of future economic evaluations to recognize potential challenges and draw backs in their study design. Moreover, it can play an important role in assessing the quality of evidence from previously conducted economic evaluations focusing on palliative and end of life care patients.

A second aim of this systematic review is to synthesize recommendations and potential solutions to overcome the identified challenges when conducting economic evaluations in the palliative or end of life care settings. These findings can help to clarify current methodological questions and present guidance for authors when setting up the methodology for future economic evaluations.

The summary of methodological challenges, which have remained unresolved so far is another aim of this systematic review and should guide researchers when planning and prioritizing evaluation

For peer review only - http://bmjopen.bmj.com/site/about/guidelines.xhtml

studies in regards to methodological questions of economic evaluations in the palliative and end of life care studies.

Further, recognizing the absence of a comprehensive framework addressing the specific challenges of designing and executing economic evaluations in the palliative or end of life care settings, the results of this systematic review may form the basis of new, setting specific methods guidelines. Given we identify sufficient information in regards to recommendations and potential solutions in respect to the challenges identified, as well as guidelines and evaluation studies in this field, we will develop a methodological framework guideline for economic evaluations in the palliative or end of life care settings in form of a critical appraisal checklist of good practice. This will add to available general reporting guidelines, such as CHEERS [41]. In the other case, we will highlight the areas, which will need further attention and outline a research agenda, in order to be able set up such a framework. With the proposed framework, it is aimed to improve and standardize the methodology and execution of future economic evaluations conducted in the palliative or end of life care settings, which should increase their comparability and overall transparency.

Our research question is quiet broad with numerous challenges in the development of a targeted search strategy without this being too narrow and missing relevant articles neither being too broad resulting in an unmanageable amount of hits. Therefore, a robust methodology including specific search strategy for multiple electronic databases of peer-reviewed literature, and tailored search strings carefully refined for every database searched and developed in cooperation with an information specialist have been developed and piloted.
Further, the screening process will be double screened by two individual researchers, considering articles published in English, German, Dutch, Spanish and Spanish as eligible for inclusion. While this language restriction might present a potential limitation, this shall be only marginal in the high income setting considering also the good coverage of countries with strong track record in economic evaluations and palliative/end of life care aspects. Consequently, it is likely that generalisability of the overall findings will be limited to Europe/North America/South America and Australia. A separate review tailored to low and middle-income settings may become necessary if the current review identifies relevant cultural/spiritual/preference aspects important also for economic evaluations. Formal transferability assessment will be carried out as far as possible.

The quality of a systematic review is highly dependent on its transparency of the methodology and reporting of results. To assure full transparency the systematic review protocol is reported according to the recommendations of the Preferred Reporting Items for Systematic Review and Meta-Analysis Protocols (PRISMA-P) statement. Registration with the International Prospective Register of Systematic Reviews (PROSPERO) is ongoing. Also the systematic review itself will follow the

For peer review only - http://bmjopen.bmj.com/site/about/guidelines.xhtml

recommendations of the Preferred Reporting Items for Systematic Review and Meta-Analysis (PRISMA) statement.

A final strength of this systematic review is that it will be carried out alongside a large EU project (the iLIVE project), which allows direct testing of our findings and develop practice-tested recommendations for this area. Further, the iLIVE project includes different relevant stakeholders, which enables a comprehensive external review of our findings. Specifically, we will ensure to involve the iLIVE PP advisory board representatives in the interpretation of the review findings.

Author affiliations

[1] Department of Health Economics, Center for Public Health, Medical University of Vienna, Kinderspitalgasse 15/1, 1090 Vienna, Austria

[2] University Library, Medical University of Vienna, Währinger Gürtel 18-20, 1090 Vienna, Austria

For peer review only

For peer review only - http://bmjopen.bmj.com/site/about/guidelines.xhtml

REFERENCES

1. Hofstede, J.M., et al., *Differences in palliative care quality between patients with cancer, patients with organ failure and frail patients: a study based on measurements with the consumer quality index palliative care for bereaved relatives.* Palliative medicine, 2016. **30**(8): p. 780-788.
2. De Flora, S., et al., *The epidemiological revolution of the 20th century.* The FASEB Journal, 2005. **19**(8): p. 892-897.
3. van der Velden, L.F., et al., *Dying from cancer or other chronic diseases in the Netherlands: ten-year trends derived from death certificate data.* BMC palliative care, 2009. **8**(1): p. 4.
4. Organization, W.H., *Palliative care for older people: better practices.* 2011.
5. Van den Block, L. and G. Albers, *Palliative care for older people: a public health perspective*. 2015: Oxford University Press, USA.
6. Higginson, I.J., *It would be NICE to have more evidence?* 2004, SAGE Publications Sage UK: London, England.
7. Scitovsky, A.A., *"The high cost of dying": what do the data show?* The Milbank Quarterly, 2005. **83**(4): p. 825.
8. Hogan, C., et al., *Medicare beneficiaries' costs of care in the last year of life.* Health affairs, 2001. **20**(4): p. 188-195.
9. Lubitz, J.D. and G.F. Riley, *Trends in Medicare payments in the last year of life.* New England journal of medicine, 1993. **328**(15): p. 1092-1096.
10. Hoffmann, F. and R. Rodrigues, *Informal carers: who takes care of them?* 2010.
11. Drummond, M.F., et al., *Methods for the economic evaluation of health care programmes*. 2015: Oxford university press.
12. Tuffaha, H.W., L.G. Gordon, and P.A. Scuffham, *Value of information analysis in healthcare: a review of principles and applications.* Journal of medical economics, 2014. **17**(6): p. 377-383.
13. Gomes, B., et al., *Optimal approaches to the health economics of palliative care: report of an international think tank.* Journal of pain and symptom management, 2009. **38**(1): p. 4-10.
14. Bausewein, C., et al., *Implementing patient reported outcome measures (PROMs) in palliative care-users' cry for help.* Health and quality of life outcomes, 2011. **9**(1): p. 27.
15. Higginson, I.J., et al., *Evaluating complex interventions in end of life care: the MORECare statement on good practice generated by a synthesis of transparent expert consultations and systematic reviews.* BMC medicine, 2013. **11**(1): p. 111.
16. Round, J., *Is a QALY still a QALY at the end of life?* Journal of Health Economics, 2012. **31**(3): p. 521-7.
17. McCaffrey, N.E., S., *Raise the Bar, Not the Threshold Value: Meeting Patient Preferences for Palliative and End-of-Life Care.* PharmacoEconomics - Open, 2018. **2**(2): p. 93-95.
18. May, P., C. Normand, and R.S. Morrison, *Economic impact of hospital inpatient palliative care consultation: review of current evidence and directions for future research.* Journal of Palliative Medicine, 2014. **17**(9): p. 1054-63.
19. Norton, E.C., *Long-term care.* Handbook of health economics, 2000. **1**: p. 955-994.
20. Dumont, S., et al., *The trajectory of palliative care costs over the last 5 months of life: a Canadian longitudinal study.* Palliative Medicine, 2010. **24**(6): p. 630-40.
21. Chai, H., et al., *The magnitude, share and determinants of unpaid care costs for home-based palliative care service provision in Toronto, Canada.* Health & Social Care in the Community, 2014. **22**(1): p. 30-9.
22. Evans, C.J., et al., *'Best practice' in developing and evaluating palliative and end-of-life care services: a meta-synthesis of research methods for the MORECare project.* Palliative Medicine, 2013. **27**(10): p. 885-98.
23. Guerriere, D.N.C., P. C., *The Ambulatory and Home Care Record: A Methodological Framework for Economic Analyses in End-of-Life Care.* Journal of Aging Research, 2011. **2011**: p. 374237.

For peer review only - http://bmjopen.bmj.com/site/about/guidelines.xhtml

24. Gardiner, C., et al., *What cost components are relevant for economic evaluations of palliative care, and what approaches are used to measure these costs? A systematic review.* Palliative Medicine, 2017. **31**(4): p. 323-337.
25. Brick, A., et al., *Costs of formal and informal care in the last year of life for patients in receipt of specialist palliative care.* Palliative Medicine, 2017. **31**(4): p. 356-368.
26. Gardiner, C., C. McDermott, and C. Hulme, *Costs of Family Caregiving in Palliative Care (COFAC) questionnaire: development and piloting of a new survey tool.* BMJ supportive & palliative care, 2019. **9**(3): p. 300-306.
27. Andersson, A., L.-Å. Levin, and B.G. Emtinger, *The economic burden of informal care.* International Journal of Technology Assessment in Health Care, 2002. **18**(1): p. 46-54.
28. Gardiner, C., et al., *Methodological considerations for researching the financial costs of family caregiving within a palliative care context.* BMJ supportive & palliative care, 2016. **6**(4): p. 445-451.
29. May, P.N., C.: Morrison, R. S., *Economic impact of hospital inpatient palliative care consultation: review of current evidence and directions for future research.* Journal of Palliative Medicine, 2014. **17**(9): p. 1054-63.
30. Moher, D., et al., *Preferred reporting items for systematic review and meta-analysis protocols (PRISMA-P) 2015 statement.* Systematic reviews, 2015. **4**(1): p. 1.
31. Beller, E.M., et al., *PRISMA for abstracts: reporting systematic reviews in journal and conference abstracts.* PLoS medicine, 2013. **10**(4): p. e1001419.
32. van Mastrigt, G.A., et al., *How to prepare a systematic review of economic evaluations for informing evidence-based healthcare decisions: a five-step approach (part 1/3).* Expert review of pharmacoeconomics & outcomes research, 2016. **16**(6): p. 689-704.
33. Dy, S.M., et al., *Closing the quality gap: revisiting the state of the science (vol. 8: improving health care and palliative care for advanced and serious illness).* Evidence report/technology assessment, 2012(2088): p. 1.
34. Rietjens, J.A., et al., *Development and validation of search filters to find articles on palliative care in bibliographic databases.* Palliative medicine, 2019. **33**(4): p. 470-474.
35. Starr, L.T., et al., *Associations Among End-of-Life Discussions, Health-Care Utilization, and Costs in Persons With Advanced Cancer: A Systematic Review.* American Journal of Hospice and Palliative Medicine®, 2019: p. 1049909119848148.
36. Sassi, F., L. Archard, and D. McDaid, *Searching literature databases for health care economic evaluations: how systematic can we afford to be?* Medical care, 2002. **40**(5): p. 387-394.
37. McKinlay, R.J., N.L. Wilczynski, and R.B. Haynes, *Optimal search strategies for detecting cost and economic studies in EMBASE.* BMC Health Services Research, 2006. **6**(1): p. 67.
38. Gomes, B., et al., *Effectiveness and cost ‐ effectiveness of home palliative care services for adults with advanced illness and their caregivers.* Cochrane Database of Systematic Reviews, 2013(6).
39. Organization", W.H. *Definition of key terms* 2020 07.01.2020]; Available from: https://www.who.int/hiv/pub/guidelines/arv2013/intro/keyterms/en/.
40. Ryan, R., *Cochrane Consumers and Communication Review Group: data synthesis and analysis.* Cochrane Consumers and Communication Review Group, available at: http://cccrg. cochrane. org [Accessed 8 March 2016].[Google Scholar], 2013.
41. Husereau, D., et al., *Consolidated health economic evaluation reporting standards (CHEERS)—explanation and elaboration: a report of the ISPOR health economic evaluation publication guidelines good reporting practices task force.* Value in Health, 2013. **16**(2): p. 231-250.
42. Wijnen, B.F., et al., *How to prepare a systematic review of economic evaluations for informing evidence-based healthcare decisions: data extraction, risk of bias, and transferability (part 3/3).* Expert review of pharmacoeconomics & outcomes research, 2016. **16**(6): p. 723-732.

For peer review only

43. Excellence, N.-N.I.f.H.a.C. *Methods for the development of NICE public health guidance (third edition)*. 2012 accessed 07.01.2020]; Available from: https://www.nice.org.uk/process/pmg4/chapter/appendix-f-quality-appraisal-checklist-quantitative-intervention-studies.

44. Moher, D., et al., *Preferred reporting items for systematic reviews and meta-analyses: the PRISMA statement.* Annals of internal medicine, 2009. **151**(4): p. 264-269.

45. Puts, M.T.E., et al., *Clinical and Cost-effectiveness of a Comprehensive geriatric assessment and management for Canadian elders with Cancer-the 5C study: a study protocol for a randomised controlled phase III trial.* BMJ Open, 2019. **9**(5): p. e024485.

AUTHORS' CONTRIBUTIONS

JS and CF conceived of the idea, developed the research question, study methods and data extraction form. EC and CF designed and developed the search strategy and strings. CF drafted the manuscript with contribution from JS. All authors approved the final manuscript.

FUNDING STATEMENT

This systematic review is part of the ‘iLIVE project –live well, die well, a research programme to support living until the end` which has received funding from the European Union's Horizon 2020 research and innovation programme under grant agreement No 825731. The funders did not play a role in decisions in the development or publishing of the protocol.

PATIENT CONSENT FOR PUBLICATION

Not required.

COMPETING INTERESTS STATEMENT

The authors declare that they have no conflict of interest.

For peer review only

For peer review only - http://bmjopen.bmj.com/site/about/guidelines.xhtml

APPENDIX

Appendix 1

PRISMA-P (Preferred Reporting Items for Systematic review and Meta-Analysis Protocols) 2015 checklist: recommended items to address in a systematic review protocol*

Section and topic	Item No	Checklist item	Reported
**ADMINISTRATIVE INFORMATION**			
Title:			
Identification	1a	Identify the report as a protocol of a systematic review	Title page
Update	1b	If the protocol is for an update of a previous systematic review, identify as such	N/A
Registration	2	If registered, provide the name of the registry (such as PROSPERO) and registration number	Page 2
Authors:			
Contact	3a	Provide name, institutional affiliation, e-mail address of all protocol authors; provide physical mailing address of corresponding author	Title page
Contributions	3b	Describe contributions of protocol authors and identify the guarantor of the review	Page 10
Amendments	4	If the protocol represents an amendment of a previously completed or published protocol, identify as such and list changes; otherwise, state plan for documenting important protocol amendments	N/A
Support:			
Sources	5a	Indicate sources of financial or other support for the review	Page 10
Sponsor	5b	Provide name for the review funder and/or sponsor	Page 10
Role of sponsor or funder	5c	Describe roles of funder(s), sponsor(s), and/or institution(s), if any, in developing the protocol	Page 10
**INTRODUCTION**			
Rationale	6	Describe the rationale for the review in the context of what is already known	Page 3
Objectives	7	Provide an explicit statement of the question(s) the review will address with reference to participants, interventions, comparators, and outcomes (PICO)	Page 3-4
**METHODS**			
Eligibility criteria	8	Specify the study characteristics (such as PICO, study design, setting, time frame) and report characteristics (such as years considered, language, publication status) to be used as criteria for eligibility for the review	Page 4-6

For peer review only

Information sources	9	Describe all intended information sources (such as electronic databases, contact with study authors, trial registers or other grey literature sources) with planned dates of coverage	Page 5
Search strategy	10	Present draft of search strategy to be used for at least one electronic database, including planned limits, such that it could be repeated	Page 6-7 Appendix 2
Study records:			
Data management	11a	Describe the mechanism(s) that will be used to manage records and data throughout the review	Page 6
Selection process	11b	State the process that will be used for selecting studies (such as two independent reviewers) through each phase of the review (that is, screening, eligibility and inclusion in meta-analysis)	Page 7-8
Data collection process	11c	Describe planned method of extracting data from reports (such as piloting forms, done independently, in duplicate), any processes for obtaining and confirming data from investigators	Page 8
Data items	12	List and define all variables for which data will be sought (such as PICO items, funding sources), any pre-planned data assumptions and simplifications	Page 4-6
Outcomes and prioritization	13	List and define all outcomes for which data will be sought, including prioritization of main and additional outcomes, with rationale	Page 4 and 6
Risk of bias in individual studies	14	Describe anticipated methods for assessing risk of bias of individual studies, including whether this will be done at the outcome or study level, or both; state how this information will be used in data synthesis	Page 9
Data synthesis	15a	Describe criteria under which study data will be quantitatively synthesised	Page 8
	15b	If data are appropriate for quantitative synthesis, describe planned summary measures, methods of handling data and methods of combining data from studies, including any planned exploration of consistency (such as I^2, Kendall's τ)	N/A
	15c	Describe any proposed additional analyses (such as sensitivity or subgroup analyses, meta-regression)	N/A
	15d	If quantitative synthesis is not appropriate, describe the type of summary planned	N/A
Meta-bias(es)	16	Specify any planned assessment of meta-bias(es) (such as publication bias across studies, selective reporting within studies)	N/A
Confidence in cumulative evidence	17	Describe how the strength of the body of evidence will be assessed (such as GRADE)	Page 9

* It is strongly recommended that this checklist be read in conjunction with the PRISMA-P Explanation and Elaboration (cite when available) for important clarification on the items. Amendments to a review protocol should be tracked and dated. The copyright for PRISMA-P (including checklist) is held by the PRISMA-P Group and is distributed under a Creative Commons Attribution Licence 4.0.

For peer review only

The PRISMA for Abstracts Checklist

TITLE	CHECKLIST ITEM	REPORTED ON PAGE #
**1.Title**	Identify the report as a systematic review, meta-analysis, or both.	2 (Introduction)
**BACKGROUND**		
**2.Objectives:**	The research question including components such as participants, interventions, comparators, and outcomes.	2 (Introduction)
**METHODS**		
**3.Eligibility criteria:**	Study and report characteristics used as criteria for inclusion.	2 (Introduction)
**4.Information sources:**	Key databases searched and search dates.	2 (Methods and analysis)
**5. Risk of bias:**	Methods of assessing risk of bias.	2 (Methods and analysis)
**RESULTS**		
**6. Included studies:**	Number and type of included studies and participants and relevant characteristics of studies.	n.a.
**7. Synthesis of results:**	Results for main outcomes (benefits and harms), preferably indicating the number of studies and participants for each. If meta-analysis was done, include summary measures and confidence intervals.	n.a.
**8. Description of the effect:**	Direction of the effect (i.e. which group is favoured) and size of the effect in terms meaningful to clinicians and patients.	n.a.
**DISCUSSION**		
**9. Strengths and Limitations of evidence:**	Brief summary of strengths and limitations of evidence (e.g. inconsistency, imprecision, indirectness, or risk of bias, other supporting or conflicting evidence)	n.a.
**10. Interpretation:**	General interpretation of the results and important implications	n.a.
**OTHER**		
**11. Funding:**	Primary source of funding for the review.	2 (Acknowledgements)
**12. Registration:**	Registration number and registry name.	2 (Methods and analysis)

Appendix 2

Search syntax in different databases

EMBASE.com

#33 #28 NOT #31 AND [1-1-1999]/sd NOT [15-11-2019]

#32 #28 NOT #31

#31 #29 NOT #30

#30 #29 AND ([young adult]/lim OR [adult]/lim OR [middle aged]/lim OR [aged]/lim OR [very elderly]/lim)

#29 #28 AND ([adolescent]/lim OR [child]/lim OR [fetus]/lim OR [infant]/lim OR [newborn]/lim OR [preschool]/lim OR [school]/lim)

#28 #23 NOT #26 AND ([dutch]/lim OR [english]/lim OR [french]/lim OR [german]/lim OR [spanish]/lim)

#27 #23 NOT #26

#26 #24 OR #25

#25 waste:jt OR 'life cycle assessment':jt

#24 recycl*:ti,de,kw,jt

#23 #13 AND #22

#22 #14 OR #15 OR #16 OR #17 OR #18 OR #19 OR #20 OR #21

#21 (('health care' OR healthcare) NEAR/3 cost*):ti,kw

#20 economics:ti,kw

#19 (economic* NEAR/3 (evaluat* OR aspect* OR health OR analy* OR model* OR framework* OR 'frame work*' OR method*)):ti,ab,kw

#18 'health care cost'/exp/mj

#17 'economics'/mj

#16 'economic model'/de

#15 'health economics'/de

For peer review only

#14 'economic evaluation'/de

#13 #1 OR #2 OR #3 OR #4 OR #5 OR #6 OR #7 OR #8 OR #9 OR #10 OR #11 OR #12

#12 bereave*:de,ti,ab,kw

#11 hospice*:de,ti,ab,kw

#10 'advanced cancer':ti,ab,kw

#9 'end of life':de,ti,ab,kw OR 'last year of life':de,ti,ab,kw OR 'lyol':de,ti,ab,kw OR 'life s end':de,ti,ab,kw

#8 (terminal* NEAR/6 (care* OR caring OR ill OR illness* OR patient*)):ti,ab,kw

#7 palliat*:ti,ab,kw

#6 'hospice'/de

#5 'terminal disease'/de

#4 'palliative therapy'/exp

#3 'terminal care'/exp

#2 'terminally ill patient'/exp

#1 'palliative nursing'/de

EBM Reviews - Health Technology Assessment Database (OVID) (discontinued at the end of 2016)

1 Palliative Care/

2 exp Terminal Care/

3 terminally ill/

4 palliat*.mp.

5 (terminal* adj6 (care or caring or ill or illness*)).mp.

6 (end of life or last year of life or lyol or life* end).mp.

7 advanced cancer.mp.

8 hospices/

9 hospice*.mp.

For peer review only

10 bereave*.mp.

11 1 or 2 or 3 or 4 or 5 or 6 or 7 or 8 or 9 or 10

12 exp Health Care Costs/

13 ((health care or healthcare) adj3 cost*).mp.

14 "Costs and Cost Analysis"/

15 Cost-Benefit Analysis/mt [Methods]

16 exp models, economic/

17 (economic* adj3 (evaluat* or aspect* or health or analy* or model* or framework* or frame work* or method*)).mp.

18 economics.mp.

19 12 or 13 or 14 or 15 or 16 or 17 or 18

20 11 and 19

21 limit 20 to "all child (0 to 18 years)"

22 limit 20 to (dutch or english or german or spanish or french)

EBM Reviews - NHS Economic Evaluation Database (OVID) (discontinued at the 31st March 2015)

1 Palliative Care/

2 exp Terminal Care/

3 terminally ill/

4 palliat*.mp. [mp=title, text, subject heading word]

5 (terminal* adj6 (care or caring or ill or illness*)).mp. [mp=title, text, subject heading word]

6 (end of life or last year of life or lyol or life* end).mp. [mp=title, text, subject heading word]

7 advanced cancer.mp. [mp=title, text, subject heading word]

8 hospices/

9 hospice*.mp. [mp=title, text, subject heading word]

10 bereave*.mp. [mp=title, text, subject heading word]

11 1 or 2 or 3 or 4 or 5 or 6 or 7 or 8 or 9 or 10

For peer review only

12 limit 11 to "all child (0 to 18 years)"

13 limit 12 to "all adult (19 plus years)"

14 12 not 13

15 11 not 14

16 limit 15 to yr="1999 - 2015"

For peer review only

Appendix 3

Data extraction form: methodological studies

**Reviewer:**	…………………………………	**Date:**	…………………………………
**Author:**	…………………………………	**Country:**	…………………………………
**Reference type:**	…………………………………	**Manuscript code:**	…………………………………
**Name of journal/book chapter/website etc. :**	…………………………………	**Language of publication**	…………………………………
**Date of publication:**	…………………………………	**Page number:**	…………………………………
**Study design:**			
Case report	O	Review (scoping, systematic, narrative)	O
Economic evaluation	O	Guideline or checklist	O
Editorial/commentary/letter:	O	Other	O
Cohort	O		
**Quality appraisal score:**			
Quality appraisal applied:	…………………………………	Quality rating:	…………………………………
**Aspect of economic evaluation (EE) described:**			
O Study population	O Study perspective	O Time horizon	O Type of EE
O Outcomes	O Data (sources)	O Costs	O Resource use measurement
O Valuation method	O Adjustments for inflation/deflation	O Discounting	O Ethical implications
O Decision modelling	O Care setting	O Other: ……………………………………………	

For peer review only

Data extraction form: applied economic evaluations

**Reviewer:**	…………………………………	**Date:**	…………………………………
**Author:**	…………………………………	**Manuscript code:**	…………………………………
**Name of journal:**	…………………………………	**Language of publication**	…………………………………
**Date of publication:**	…………………………………		
**Study characteristics:**			
Funding source (p.n.*)	…………………………………	Year of analysis (p.n.):	…………………………………
Study design (p.n.):	…………………………………	Type of economic evaluation (CEA, CUA. etc) (p.n.):	…………………………………
Author stated analytical perspective (p.n.):	…………………………………	Time frame of analysis (p.n.):	…………………………………
Country (p.n.):	…………………………………	Study sector (hospice, home, hospital etc. (p.n.):	…………………………………
Modelling used (p.n.):	…………………………………	Quality appraisal score (Quality appraisal):	…………………………………
Intervention description:	…………………………………		
**Patient cohort characteristics:**			
Control group size:	…………………………………	Intervention group size:	…………………………………
Target population definiton:	…………………………………		
**Methods :**			
Analysis of uncertainty:	…………………………………		
**Outcomes :**			
Outcomes measured:	…………………………………	Discounting:	…………………………………
Data sources :	…………………………………		
**Costs:**			
Cost categories included:	…………………………………	Discounting:	…………………………………

For peer review only

Unit cost sources:	…………………………….	Resource use info source:	…………………………….
Reviewer stated analytical perspective:	…………………………….	Year of costing:	…………………………….
Adjustments for inflation/deflation:	…………………………….		
**Cost-effectiveness:**			
Main CE results:	…………………………….	Analysis of uncertainty:	…………………………….

*p.n. - page number

For peer review only

PRISMA-P (Preferred Reporting Items for Systematic review and Meta-Analysis Protocols) 2015 checklist: recommended items to address in a systematic review protocol*

Section and topic	Item No	Checklist item	Reported
**ADMINISTRATIVE INFORMATION**			
Title:			
Identification	1a	Identify the report as a protocol of a systematic review	Title page
Update	1b	If the protocol is for an update of a previous systematic review, identify as such	N/A
Registration	2	If registered, provide the name of the registry (such as PROSPERO) and registration number	Page 2
Authors:			
Contact	3a	Provide name, institutional affiliation, e-mail address of all protocol authors; provide physical mailing address of corresponding author	Title page
Contributions	3b	Describe contributions of protocol authors and identify the guarantor of the review	Page 10
Amendments	4	If the protocol represents an amendment of a previously completed or published protocol, identify as such and list changes; otherwise, state plan for documenting important protocol amendments	N/A
Support:			
Sources	5a	Indicate sources of financial or other support for the review	Page 10
Sponsor	5b	Provide name for the review funder and/or sponsor	Page 10
Role of sponsor or funder	5c	Describe roles of funder(s), sponsor(s), and/or institution(s), if any, in developing the protocol	Page 10
**INTRODUCTION**			
Rationale	6	Describe the rationale for the review in the context of what is already known	Page 3
Objectives	7	Provide an explicit statement of the question(s) the review will address with reference to participants, interventions, comparators, and outcomes (PICO)	Page 3-4
**METHODS**			
Eligibility criteria	8	Specify the study characteristics (such as PICO, study design, setting, time frame) and report characteristics (such as years considered, language, publication status) to be used as criteria for eligibility for the review	Page 4-6
Information sources	9	Describe all intended information sources (such as electronic databases, contact with study authors, trial registers or other grey literature sources) with planned dates of coverage	Page 5
Search strategy	10	Present draft of search strategy to be used for at least one electronic database, including planned limits, such that it could be repeated	Page 6-7 Appendix 2

For peer review only

Study records:			
Data management	11a	Describe the mechanism(s) that will be used to manage records and data throughout the review	Page 6
Selection process	11b	State the process that will be used for selecting studies (such as two independent reviewers) through each phase of the review (that is, screening, eligibility and inclusion in meta-analysis)	Page 7-8
Data collection process	11c	Describe planned method of extracting data from reports (such as piloting forms, done independently, in duplicate), any processes for obtaining and confirming data from investigators	Page 8
Data items	12	List and define all variables for which data will be sought (such as PICO items, funding sources), any pre-planned data assumptions and simplifications	Page 4-6
Outcomes and prioritization	13	List and define all outcomes for which data will be sought, including prioritization of main and additional outcomes, with rationale	Page 4 and 6
Risk of bias in individual studies	14	Describe anticipated methods for assessing risk of bias of individual studies, including whether this will be done at the outcome or study level, or both; state how this information will be used in data synthesis	Page 9
Data synthesis	15a	Describe criteria under which study data will be quantitatively synthesised	Page 8
	15b	If data are appropriate for quantitative synthesis, describe planned summary measures, methods of handling data and methods of combining data from studies, including any planned exploration of consistency (such as I^2, Kendall's τ)	N/A
	15c	Describe any proposed additional analyses (such as sensitivity or subgroup analyses, meta-regression)	N/A
	15d	If quantitative synthesis is not appropriate, describe the type of summary planned	N/A
Meta-bias(es)	16	Specify any planned assessment of meta-bias(es) (such as publication bias across studies, selective reporting within studies)	N/A
Confidence in cumulative evidence	17	Describe how the strength of the body of evidence will be assessed (such as GRADE)	Page 9

* It is strongly recommended that this checklist be read in conjunction with the PRISMA-P Explanation and Elaboration (cite when available) for important clarification on the items. Amendments to a review protocol should be tracked and dated. The copyright for PRISMA-P (including checklist) is held by the PRISMA-P Group and is distributed under a Creative Commons Attribution Licence 4.0.

For peer review only

BMJ Open

Methodologic aspects of economic evaluations conducted in the palliative or end of life care settings: a systematic review protocol

Journal:	*BMJ Open*
Manuscript ID	bmjopen-2019-035760.R2
Article Type:	Protocol
Date Submitted by the Author:	17-Mar-2020
Complete List of Authors:	Fischer, Claudia; Medical University of Vienna Center for Public Health, Health Economics<br>Chwala, Eva; Medical University of Vienna, University Library<br>Simon, Judit; Medical University of Vienna Center for Public Health, Health Economics
**Primary Subject Heading**:	Palliative care
Secondary Subject Heading:	Palliative care, Research methods, Health economics
Keywords:	PALLIATIVE CARE, STATISTICS & RESEARCH METHODS, HEALTH ECONOMICS, Adult palliative care < PALLIATIVE CARE

SCHOLARONE™
Manuscripts

BMJ

I, the Submitting Author has the right to grant and does grant on behalf of all authors of the Work (as defined in the below author licence), an exclusive licence and/or a non-exclusive licence for contributions from authors who are: i) UK Crown employees; ii) where BMJ has agreed a CC-BY licence shall apply, and/or iii) in accordance with the terms applicable for US Federal Government officers or employees acting as part of their official duties; on a worldwide, perpetual, irrevocable, royalty-free basis to BMJ Publishing Group Ltd ("BMJ") its licensees and where the relevant Journal is co-owned by BMJ to the co-owners of the Journal, to publish the Work in this journal and any other BMJ products and to exploit all rights, as set out in our licence.

The Submitting Author accepts and understands that any supply made under these terms is made by BMJ to the Submitting Author unless you are acting as an employee on behalf of your employer or a postgraduate student of an affiliated institution which is paying any applicable article publishing charge ("APC") for Open Access articles. Where the Submitting Author wishes to make the Work available on an Open Access basis (and intends to pay the relevant APC), the terms of reuse of such Open Access shall be governed by a Creative Commons licence – details of these licences and which Creative Commons licence will apply to this Work are set out in our licence referred to above.

Other than as permitted in any relevant BMJ Author's Self Archiving Policies, I confirm this Work has not been accepted for publication elsewhere, is not being considered for publication elsewhere and does not duplicate material already published. I confirm all authors consent to publication of this Work and authorise the granting of this licence.

For peer review only

Methodologic aspects of economic evaluations conducted in the palliative or end of life care settings: a systematic review protocol

Claudia Fischer[1], Eva Chwala[2], Judit Simon[1]

Corresponding author: Claudia Fischer[1]

E-mail address of corresponding author: Claudia.fischer@meduniwien.ac.at

For peer review only

For peer review only - http://bmjopen.bmj.com/site/about/guidelines.xhtml

ABSTRACT

Introduction: In light of this growing palliative care and end of life care patient population, as well as new (expensive) drugs and treatments, quality research providing evidence for decision-making is required. However, common research guidance is lacking in this field, especially in respect to the methods applied in economic evaluations. Therefore, the aim of the planned systematic review is to identify and summarize relevant information on methodological challenges, potential solutions and recommendations for conducting economic evaluations of interventions in adult patients, irrespective of their underlying disease and gender in the palliative or end of life care settings, with no restrictions in regards to countries/geographic regions. The results of this systematic review may help to clarify the current methodological questions and form the basis of new, setting specific methods guidelines and support ongoing applied economic evaluations in the field.

Methods and analysis: A systematic review will be conducted using MEDLINE, EMBASE, Health Technology Assessment Database, and NHS Economic Evaluation Database to identify studies published from 1999 onwards with relevant information on methodological challenges, potential solutions, and recommendations for conducting economic evaluations in the palliative or end of life care settings. Articles in English, German, Spanish, French or Dutch language will be considered. Two independent reviewers will conduct the screening of articles; any discrepancies will be resolved by discussion and involvement of a third reviewer. Pre-designed data extraction forms will be applied, consequently narratively synthesized and categorized. Studies' methodological quality will be critically appraised. Besides existing economic guidelines and checklists for specific information on the palliative and end of life care sector will be searched. The International Prospective Register of Systematic Reviews (PROSPERO) registration number is CRD42020148160.

Ethics and dissemination: Ethical approval is not required, as this is a planned systematic review of published literature. An article will be disseminated in a related peer-reviewed journal, as well as presented at leading palliative care and health economic conferences.

Acknowledgments: The `iLIVE project – live well, die well, a research programme to support living until the end´ has received funding from the European Union’s Horizon 2020 research and innovation programme under grant agreement No 825731.

For peer review only - http://bmjopen.bmj.com/site/about/guidelines.xhtml

STRENGTHS AND LIMITATIONS OF THIS STUDY

- To our knowledge, this systematic review, which will be conducted and reported according to the current highest methodological standard, is the first one to identify methodological aspects and challenges to consider when conducting an economic evaluation in the palliative or end of life care settings.
- A robust methodology including specific search strategy, tailored and pilot tested search strings developed in cooperation with an information specialist, has been applied to develop a targeted search for our broad research question
- While our language restriction in the screening process might present a potential limitation, this shall be only marginal in the high-income setting considering also the good coverage of countries with a strong track record in economic evaluations and palliative/end of life care aspects.
- Although formal transferability assessment will be carried out as far as possible, a separate review tailored to low and middle-income settings may become necessary if the current review identifies relevant cultural/spiritual/preference aspects important also for economic evaluations.
- This systematic review will be carried out alongside a large EU project (the iLIVE project), which allows direct testing of our findings, develop practice-applied recommendations and external review of our findings by the iLIVE stakeholders.

For peer review only

For peer review only - http://bmjopen.bmj.com/site/about/guidelines.xhtml

INTRODUCTION

Over the past century, patterns of mortality in the Western world have changed [1]. While sudden death, mainly due to infectious disease, used to be the major cause of death, nowadays there is an increase of the ageing population living with advanced stages of incurable chronic conditions such as cancer, chronic heart failure or chronic obstructive pulmonary disease (COPD) near the end of life [2, 3].

It is crucial to enable this growing patient group to live well at the end of their lives as well as to die well [4, 5]. This requires quality research providing evidence for decisions on clinical guidelines, treatments and services [6]. In light of the new (expensive) drugs and interventions, an effective and efficient resource allocation is of great importance in the palliative and end of life care setting [7]. Studies have estimated the cost of care in the last year of life at 25 to 30% of all medical expenditures during a lifetime [8, 9]. This number is thought to even further increase in light of the ageing population and the prospect of the potentially reduced number of informal carers in the future [10].

Economic evaluations are the comparative analysis of alternative courses of action in terms of both, their costs and consequences [11], and have been widely applied in other fields to answer questions in regards to the cost-effectiveness of health care interventions and inform decision-makers and commissioners [12]. Nevertheless, the economic component has often been overlooked in palliative care or end of life care studies, leaving several unanswered questions for the quality assurance, monitoring, funding, and evaluation of palliative care or end of life care [13, 14]. Palliative and end of life care research lack common research guidance [15], which would be essential as these settings differ from other health care fields in several aspects, which is why existing methods of economic evaluations may not be suitable for them. For example, compared to interventions in other fields, the ultimate focus of interventions in the palliative or end of life care settings is not on the extension of life but rather on the quality of dying, patient's dignity or strengthening relationships with loved ones [16, 17]. Therefore, the appropriateness of generic quality of life (QALY) measures for economic evaluations in the palliative or end of life care settings may be questioned, as their measured quality dimensions may not reflect those dimensions relevant for palliative or end of life care patients [18].

Further, it is well known that informal carers play an important role in the care of patients in many disease fields, such as chronic conditions [19]. The contribution of informal carers in the care of patients in the palliative or end of life care fields is thought to be substantially high [20, 21]. Although the general acknowledgment of their importance, often their contributions, both in respect to costs and outcomes, are not included in economic evaluations [22, 23]. This is due to diverse reasons, for

For peer review only - http://bmjopen.bmj.com/site/about/guidelines.xhtml

instance, 1) payers want a perspective applied that reflects costs that emerge for them [24], 2) there is no gold standard how to value (i.e. attribute a monetary value to) indirect costs of informal care [25-27], 3) ethical concerns about involving participants in data assessment and research at a suitable time, especially in regards to finances, which may add to the already enormous emotional and physical burden they face [28]. However, leaving these costs and outcomes unconsidered, may result in a biased reflection of the true costs and benefits of interventions in the palliative or end of life care settings [29].

To generate valid und useful evidence and decision base, the research methods of economic evaluations applied in the palliative care or end of life care fields should consider these specific conditions in their methodology. Therefore, this systematic review aims to summarize the methodological aspects and challenges (e.g. valuation of informal care, the usability of QALYs or other outcome types in this field) and recommendations as well as potential solutions to overcome these problems and consequently provide methodological guidance when conducting economic evaluations in the palliative or end of life care settings, irrespective of the underlying disease, gender or geographic region.

OBJECTIVES

The specific objectives of this study are as follows:

1. To identify methodological aspects and challenges of conducting economic evaluations in palliative or end of life care patients irrespective of their underlying disease, gender or geographic region;
1. To synthesize recommendations and potential solutions to overcome these identified challenges;
2. To sum-up methodological challenges, which have remained unresolved so far; and
3. If feasible, to develop a methodological framework guideline for economic evaluations in the palliative and end of life care settings;

For peer review only - http://bmjopen.bmj.com/site/about/guidelines.xhtml

METHODS AND ANALYSIS

Protocol

This systematic review protocol has been developed according to the recommendations from the Preferred Reporting Items for Systematic Review and Meta-Analysis Protocols (PRISMA-P) 2015 statement [30] and PRISMA for Abstracts checklist [31]. A PRISMA-P file and PRISMA for Abstracts checklist is provided in online supplementary appendix 1. Besides, the review will follow the 5-step approach on the state-of-the-art methodology for conducting systematic reviews of economic evidence by Van Mastrigt et al. (2016) [32].

The work on the described systematic review has been started in June 2019 (conceptualization of the idea of the systematic review) and will take approximately until June 2021 (finalization of the data synthesis and write up of results).

Eligibility criteria

Population

Adult (a person older than 19 years of age) palliative or end of life patients, irrespective of the underlying disease, gender.

Comparator(s)

Not specified.

Context

Palliative and end of life care in different care settings such as hospitals, hospices, nursing homes, or home of the patient. No explicit restrictions in regards to countries/geographic regions are made. However, due to some necessary language restrictions, it is likely that the generalisability of the overall findings will be limited to Europe/North America/South America and Australia. Context specificity and transferability will be further assessed based on the findings.

Main outcome(s)

For peer review only - http://bmjopen.bmj.com/site/about/guidelines.xhtml

For peer review only

The outcome of interest are methodological aspects and challenges (e.g. valuation of informal care, the usability of QALYs or other outcome types in this field) for conducting economic evaluations in the palliative or end of life care settings.

Study design

To serve the broad nature of our research question, we have decided a priori to develop a protocol that includes different study types published in peer-reviewed journals in this systematic review. Articles reporting on methodological aspects and challenges of conducting economic evaluations in the palliative or end of life care setting are suspected being:

- Systematic reviews (and meta-analysis): a structured search has been applied and transparent and explicit methodological criteria are used to select the included studies.
- Narrative reviews: including a qualitative summary and discussion of pivotal studies known to the subject experts.
- Observational (e.g. case report or cohort), interventional studies.
- (Full) economic evaluations.
- Economic guidelines and checklists.
- (Editorial) discussions of the literature and commentaries: including contributions to theory building or critique, arguing the case for a field of research or a course of action, or summaries of literature.
- Qualitative studies.

Report characteristics

In this systematic review, no limit in regards to the setting will be applied, including clinical settings (e.g. hospital, nursing homes), as well as community settings or the patient's home. Depending on whether we will be able to identify sufficient information per individual setting in which care may be provided, we will present the identified information clustered per individual care setting, otherwise, no distinction per setting will be undertaken. In the field of health economics, major methodological advancements have taken place in the past 10 years, which outdate methods that were commonly applied earlier. Moreover, palliative care practice itself has undergone significant changes since 2000 [33]. For sensitivity reasons, we extended the time limit for publication one year before this date. All articles published after 1st January 1999 will be considered for inclusion. Articles written in English, German, French, Spanish or Dutch will be considered for inclusion.

For peer review only - http://bmjopen.bmj.com/site/about/guidelines.xhtml

The authors can read and analyze articles in English, German, and Dutch. Further, the iLIVE project consortium includes native speakers who will provide help with articles of interest in Spanish and French.

Information sources and search strategy

In collaboration with an information specialist, a systematic search in the electronic databases MEDLINE, EMBASE, NHS Economic Evaluation Database and the Health Technology Assessment Database produced by the Centre for Reviews and Dissemination will be systematically conducted. Appropriate Medical Subject Headings (MeSH) terms will be selected and utilized and combined with free text words to develop a search strategy, which will then be translated to the different databases. The developed search strategy will be based on a review of published search filters for palliative care and health economics (CADTH economic search filter https://www.cadth.ca/resources/finding-evidence/strings-attached-cadths-database-search-filters#eco, OVID Expert Searches Health Science http://resourcecenter.ovid.com/site/resources/expert_search/healthexp.html, CareSearch Palliative Care search filter https://www.caresearch.com.au/caresearch/tabid/377/Default.aspx, PubMed Health Sciences Research Queries https://www.nlm.nih.gov/nichsr/hedges/HSR_queries_table.html, SIGN Search filter https://www.sign.ac.uk/search-filters.html, McMaster Search filter https://hiru.mcmaster.ca/hiru/HIRU_Hedges_MEDLINE_Strategies.aspx) [34-38]. Different combinations of search terms and specifications have been tested in regard to their sensitivity for our research question. For each applied modification, the first 100 articles have been evaluated to decide whether the modification is applied or not.

Detailed inclusion and exclusion criteria are presented in Table 1.

Table 1 Inclusion and exclusion criteria

	Inclusion criteria	Exclusion criteria
Population	Adult* palliative or end of life patients, irrespective of the underlying disease, gender.	
Study design	Systematic reviews (and meta-analysis), narrative reviews, observational or interventional studies, (editorial) discussions and commentaries, (full) economic evaluations, economic guidelines and checklists, qualitative studies.	

For peer review only - http://bmjopen.bmj.com/site/about/guidelines.xhtml

Outcome	Methodological aspects and recommendations for conducting economic evaluations in the palliative and end of life care settings.	
Type of publication	Articles with available full text in English, German, Spanish, French or Dutch language.	(Conference) abstracts.

*Following the WHO definition, an adult is a person older than 19 years of age [39]

For peer review only

The final search syntax for MEDLINE (OVID) is a modification of the reviewed search strings of Gomes et al. (2016)[38] and Rietjens et al. (2019)[34]:

1 Palliative Care/
2 exp Terminal Care/
3 Terminally Ill/
4 palliat*.mp.
5 (terminal* adj6 (care or caring or ill or illness*)).ti,ab,ot,kf.
6 (end of life or last year of life or lyol or life's end).ti,ab,ot,kf.
7 advanced cancer.ti,ab,ot,kf.
8 Hospices/
9 hospice*.ti,ab,ot,kf.
10 bereave*.ti,ab,ot,kf,hw.
11 1 or 2 or 3 or 4 or 5 or 6 or 7 or 8 or 9 or 10
12 exp *Health Care Costs/
13 ((health care or healthcare) adj3 cost*).ti,ot,kf,kw.
14 *"Costs and Cost Analysis"/
15 Cost-Benefit Analysis/mt [Methods]
16 exp models, economic/
17 (economic* adj3 (evaluat* or aspect* or health or analy* or model* or framework* or frame work* or method*)).ti,ab,ot,kf,hw.
18 economics.ti,ot,kf.
19 Palliative Care/ec
20 exp Terminal Care/ec
21 Hospices/ec
22 12 or 13 or 14 or 15 or 16 or 17 or 18 or 19 or 20 or 21
23 11 and 22
24 recycl*.ti,hw,kf,jw.
25 (waste or life cycle assessment).jw.
26 24 or 25
27 23 not 26

For peer review only - http://bmjopen.bmj.com/site/about/guidelines.xhtml

28 limit 27 to (dutch or english or german or french or spanish)
29 limit 28 to "all child (0 to 18 years)"
30 limit 29 to "all adult (19 plus years)"
31 29 not 30
32 28 not 31
33 limit 32 to yr="1999 - 2019"

The search syntaxes for other databases are presented in online supplementary appendix 2.

Besides, we will screen relevant websites (standard health economic evaluation associations (e.g. ISPOR, iHEA), the resources of the members area of the International Collaborative for Best Care for the Dying Person, as well as reports of HTA bodies (e.g. NICE) to identify potential relevant guidelines to conduct economic evaluations in the palliative or end of life care settings, as well as a specific checklist for quality appraisal and value frameworks.

Furthermore, we will screen the articles for any potentially relevant article they refer to in the manuscript text.

Study records

Data management

For transparency and reproducibility a clear documentation of all searches, the electronic database searches, the references searched, and the hand search gray literature, will be kept. The search syntaxes translated for all searched databases will be made available in the appendix of the systematic review, a PRISMA flow chart will be presented, illustrating the numbers of records retrieved and selection flow through the screening rounds. All identified references will be imported and combined in a single EndNote library. Duplicate records of the same reports will be removed.

Selection process

Two screening rounds will be conducted. In the first screening round, the title and abstract of the articles will be screened. Two reviewers will independently screen all articles, the reviewers' independent decisions on inclusion and exclusion of articles will be compared and kappa statistics calculated. In case of the absence of an article's title/abstract, the full text of the article will be directly screened for potential inclusion. Those articles which are selected for further inclusion based on the title and abstract screening will undergo the second screening. In the second screening round,

For peer review only - http://bmjopen.bmj.com/site/about/guidelines.xhtml

full texts will be screened and assessed for eligibility against the pre-specified inclusion and exclusion criteria. Any disagreements will be discussed among the two, if the disagreement cannot be solved a third reviewer will be approached to reach a final decision. A PRISMA flow diagram [30] of the study selection process will be prepared to present an overview of the data collection process and the decisions that have been made in the course of it.

Data extraction

For the data extraction from papers standardized data extraction forms will be created in Excel (see appendix 3 for illustration of categories). One researcher will extract the data with the second researcher independently checking the accuracy of the extracted information. Relevant missing information or unclear information will be dealt with by contacting original authors of included studies. To ensure that the data extraction form is properly applicable, it will undergo piloting, testing it for its user-friendliness and completeness on a subsample of the total number of retrieved included studies. If questions arise, these will be discussed and if necessary, the data extraction form will be revised accordingly.

Data items

From each included article describing methodological aspects of economic evaluations in the palliative and end of life care settings standard bibliographical information (i.e. author, journal, publication year, country) and study design will be reported. Further, information on the aspect of economic evaluation described, challenge reported, the potential impact of the problem described, potential solution or recommendation described, will be abstracted. We will also collect information (e.g. study characteristics and patient characteristics) from the identified applied economic evaluation. The assessed outcomes and cost categories considered in the economic evaluation will be abstracted and summarized.

Data synthesis

A narrative synthesis of the included studies will be conducted following the Cochrane Collaboration guidelines [40] for which two data extraction sheets in Excel will be prepared, one for studies on methodological studies and one for applied economic evaluations. For the data extraction sheet for methodological studies, we a priori defined major economic evaluation characteristics by using the Consolidated Health Economic Evaluation Reporting Standards (Cheers) Checklist as a base [41]. For methodological/qualitative papers, such lists do not exist. Instead, we pre-defined a set of attributes

For peer review only - http://bmjopen.bmj.com/site/about/guidelines.xhtml

in our data extraction sheet that we will collect as standard. Of course, these categories will remain open for modification and extension, depending on which information we will identify in the data extraction and analysis phase. If required, the categories will be refined or extended depending on the identified level of information and characteristic. The level of evidence for the described challenge, potential impact, concerns and solutions will be discussed per identified sub-theme. With the help of the second data extraction sheet, which has been developed specifically for applied economic evaluations, the characteristics of the identified economic evaluations will be summarized as well as overviews of the considered outcomes and cost categories created.

For included articles, which have been executed in which the specific country and hence the specific health care system is of relevance, the transferability of study findings will be assessed using transferability checklists [42].

In case we can find sufficient information in regards to recommendations and potential solutions in respect to the challenges identified, we will develop a methodological framework guideline for economic evaluations in the palliative or end of life care setting in form of a critical appraisal checklist of good practice, aiming to improve the design, the collection of data (i.e. outcomes, and costs) as well as analysis of data in economic evaluations in this field. The methodological framework will emphasize on a set of recommendations, which differ from those when conducting economic evaluation in settings outside the palliative or end of life care settings.

The manuscript will be prepared following the Preferred Reporting Items for Systematic Reviews and Meta-Analyses (PRISMA) guidelines.

Assessment of methodological quality and risk of bias

To be able to appraise the overall quality of evidence, and the strength of a conclusion drawn from it studies methodological quality will be critically appraised. Formal quality checks will be carried out where relevant lists are readily available (e.g. NICE Quality appraisal checklists [43], CHEERS checklist [41] or PRISMA checklist [44]) and will record the quality/risk of bias information in our data extraction sheet. Two reviewers will be involved in the risk of bias assessment, one will fill in the quality appraisals, the second one will conduct quality checks, discrepancies will be solved by discussions and if necessary, consultation of a third reviewer. Once the extraction is finalized, we will decide on how this information can formally be incorporated into the synthesis of the evidence once data extraction is finalized and we have a clearer overview of the feasibility.

For peer review only - http://bmjopen.bmj.com/site/about/guidelines.xhtml

ETHICS AND DISSEMINATION

The systematic review will synthesize information described in the published literature. Ethical approval is not required, as the proposed systematic review will not use primary data. This review will provide an overview of methodological aspects to consider when conducting an economic evaluation in the palliative and end of life care settings. Findings will be widely disseminated through peer-reviewed publication, conference presentation(s) and via other planned dissemination activities in the course of the EU project 'the iLIVE project –live well, die well, a research programme to support living until the end' [45].

PATIENT AND PUBLIC INVOLVEMENT

Our results and the developed framework will be undergoing a review process, i.e. discussed and potentially refined with the experts within the iLIVE consortium and subsequently also with the palliative and end of life care community at international scientific conference discussions. Specifically, we will ensure to involve the iLIVE PP advisory board representatives, which is currently set-up and will consist of patients and public advisors, in the interpretation of the review findings. As recommended by Van Mastrigt et al (2017) these reviewers will not have been involved in the development of the framework and will include experts in the clinical area, methodological or HTA expert, or the targeted patient population [32].

DISCUSSION

To our knowledge, this will be the first systematic review, which will be conducted and reported according to the current highest methodological standard, to identify methodological aspects and challenges to consider when conducting an economic evaluation in the palliative or end of life care settings.

The systematic overview of the identified methodological aspects and challenges of conducting economic evaluations in the palliative and end of life care settings will help to deepen understanding of the challenge to conduct economic evaluations in these settings. The aim is to provide a systematic and comprehensive overview of the currently existing evidence base. For instance, provide an overview of the currently used outcome measures used in economic evaluations in the palliative and end of life care settings, provide a summary of their advantages and disadvantages,

For peer review only - http://bmjopen.bmj.com/site/about/guidelines.xhtml

and the discussion whether or not generic measures may be used in these settings and provide an overview of available evaluation studies. The overview should help authors of future economic evaluations to recognize potential challenges and drawbacks in their study design. Moreover, it can play an important role in assessing the quality of evidence from previously conducted economic evaluations focusing on palliative and end of life care patients.

The second aim of this systematic review is to synthesize recommendations and potential solutions to overcome the identified challenges when conducting economic evaluations in the palliative or end of life care settings. These findings can help to clarify current methodological questions and present guidance for authors when setting up the methodology for future economic evaluations.

The summary of methodological challenges, which have remained unresolved so far is another aim of this systematic review and should guide researchers when planning and prioritizing evaluation studies in regards to methodological questions of economic evaluations in the palliative and end of life care studies.

Further, recognizing the absence of a comprehensive framework addressing the specific challenges of designing and executing economic evaluations in the palliative or end of life care settings, the results of this systematic review may form the basis of new, setting specific methods guidelines. Given we identify sufficient information in regards to recommendations and potential solutions in respect to the challenges identified, as well as guidelines and evaluation studies in this field, we will develop a methodological framework guideline for economic evaluations in the palliative or end of life care settings in form of a critical appraisal checklist of good practice. This will add to available general reporting guidelines, such as CHEERS [41]. In the other case, we will highlight the areas, which will need further attention and outline a research agenda, in order to be able to set up such a framework. With the proposed framework, it is aimed to improve and standardize the methodology and execution of future economic evaluations conducted in the palliative or end of life care settings, which should increase their comparability and overall transparency.

Our research question is quite broad with numerous challenges in the development of a targeted search strategy without this being too narrow and missing relevant articles neither being too broad resulting in an unmanageable amount of hits. Therefore, a robust methodology including a specific search strategy for multiple electronic databases of peer-reviewed literature, and tailored search strings carefully refined for every database searched and developed in cooperation with an information specialist have been developed and piloted.

Further, the screening process will be double screened by two individual researchers, considering articles published in English, German, Dutch, Spanish and Spanish as eligible for inclusion. While this

For peer review only - http://bmjopen.bmj.com/site/about/guidelines.xhtml

language restriction might present a potential limitation, this shall be only marginal in the high-income setting considering also the good coverage of countries with a strong track record in economic evaluations and palliative/end of life care aspects. Consequently, it is likely that the generalisability of the overall findings will be limited to Europe/North America/South America and Australia. A separate review tailored to low and middle-income settings may become necessary if the current review identifies relevant cultural/spiritual/preference aspects important also for economic evaluations. Formal transferability assessment will be carried out as far as possible. The quality of a systematic review is highly dependent on its transparency of the methodology and reporting of results. To assure full transparency the systematic review protocol is reported according to the recommendations of the Preferred Reporting Items for Systematic Review and Meta-Analysis Protocols (PRISMA-P) statement. Further, the systematic review is registered with the International Prospective Register of Systematic Reviews (PROSPERO) with the registration number CRD42020148160. Also the systematic review itself will follow the recommendations of the Preferred Reporting Items for Systematic Review and Meta-Analysis (PRISMA) statement.

A final strength of this systematic review is that it will be carried out alongside a large EU project (the iLIVE project), which allows direct testing of our findings and develop practice-tested recommendations for this area. Further, the iLIVE project includes different relevant stakeholders, which enables a comprehensive external review of our findings. Specifically, we will ensure to involve the iLIVE PP advisory board representatives in the interpretation of the review findings.

Author affiliations

[1] Department of Health Economics, Center for Public Health, Medical University of Vienna, Kinderspitalgasse 15/1, 1090 Vienna, Austria

[2] University Library, Medical University of Vienna, Währinger Gürtel 18-20, 1090 Vienna, Austria

For peer review only

REFERENCES

1. Hofstede, J.M., et al., *Differences in palliative care quality between patients with cancer, patients with organ failure and frail patients: a study based on measurements with the consumer quality index palliative care for bereaved relatives.* Palliative medicine, 2016. **30**(8): p. 780-788.
2. De Flora, S., et al., *The epidemiological revolution of the 20th century.* The FASEB Journal, 2005. **19**(8): p. 892-897.
3. van der Velden, L.F., et al., *Dying from cancer or other chronic diseases in the Netherlands: ten-year trends derived from death certificate data.* BMC palliative care, 2009. **8**(1): p. 4.
4. Organization, W.H., *Palliative care for older people: better practices.* 2011.
5. Van den Block, L. and G. Albers, *Palliative care for older people: a public health perspective*. 2015: Oxford University Press, USA.
6. Higginson, I.J., *It would be NICE to have more evidence?* 2004, SAGE Publications Sage UK: London, England.
7. Scitovsky, A.A., *"The high cost of dying": what do the data show?* The Milbank Quarterly, 2005. **83**(4): p. 825.
8. Hogan, C., et al., *Medicare beneficiaries' costs of care in the last year of life.* Health affairs, 2001. **20**(4): p. 188-195.
9. Lubitz, J.D. and G.F. Riley, *Trends in Medicare payments in the last year of life.* New England journal of medicine, 1993. **328**(15): p. 1092-1096.
10. Hoffmann, F. and R. Rodrigues, *Informal carers: who takes care of them?* 2010.
11. Drummond, M.F., et al., *Methods for the economic evaluation of health care programmes*. 2015: Oxford university press.
12. Tuffaha, H.W., L.G. Gordon, and P.A. Scuffham, *Value of information analysis in healthcare: a review of principles and applications.* Journal of medical economics, 2014. **17**(6): p. 377-383.
13. Gomes, B., et al., *Optimal approaches to the health economics of palliative care: report of an international think tank.* Journal of pain and symptom management, 2009. **38**(1): p. 4-10.
14. Bausewein, C., et al., *Implementing patient reported outcome measures (PROMs) in palliative care-users' cry for help.* Health and quality of life outcomes, 2011. **9**(1): p. 27.
15. Higginson, I.J., et al., *Evaluating complex interventions in end of life care: the MORECare statement on good practice generated by a synthesis of transparent expert consultations and systematic reviews.* BMC medicine, 2013. **11**(1): p. 111.
16. Round, J., *Is a QALY still a QALY at the end of life?* Journal of Health Economics, 2012. **31**(3): p. 521-7.
17. McCaffrey, N.E., S., *Raise the Bar, Not the Threshold Value: Meeting Patient Preferences for Palliative and End-of-Life Care.* PharmacoEconomics - Open, 2018. **2**(2): p. 93-95.
18. May, P., C. Normand, and R.S. Morrison, *Economic impact of hospital inpatient palliative care consultation: review of current evidence and directions for future research.* Journal of Palliative Medicine, 2014. **17**(9): p. 1054-63.
19. Norton, E.C., *Long-term care.* Handbook of health economics, 2000. **1**: p. 955-994.
20. Dumont, S., et al., *The trajectory of palliative care costs over the last 5 months of life: a Canadian longitudinal study.* Palliative Medicine, 2010. **24**(6): p. 630-40.
21. Chai, H., et al., *The magnitude, share and determinants of unpaid care costs for home-based palliative care service provision in Toronto, Canada.* Health & Social Care in the Community, 2014. **22**(1): p. 30-9.
22. Evans, C.J., et al., *'Best practice' in developing and evaluating palliative and end-of-life care services: a meta-synthesis of research methods for the MORECare project.* Palliative Medicine, 2013. **27**(10): p. 885-98.
23. Guerriere, D.N.C., P. C., *The Ambulatory and Home Care Record: A Methodological Framework for Economic Analyses in End-of-Life Care.* Journal of Aging Research, 2011. **2011**: p. 374237.

For peer review only - http://bmjopen.bmj.com/site/about/guidelines.xhtml

24. Gardiner, C., et al., *What cost components are relevant for economic evaluations of palliative care, and what approaches are used to measure these costs? A systematic review.* Palliative Medicine, 2017. **31**(4): p. 323-337.
25. Brick, A., et al., *Costs of formal and informal care in the last year of life for patients in receipt of specialist palliative care.* Palliative Medicine, 2017. **31**(4): p. 356-368.
26. Gardiner, C., C. McDermott, and C. Hulme, *Costs of Family Caregiving in Palliative Care (COFAC) questionnaire: development and piloting of a new survey tool.* BMJ supportive & palliative care, 2019. **9**(3): p. 300-306.
27. Andersson, A., L.-Å. Levin, and B.G. Emtinger, *The economic burden of informal care.* International Journal of Technology Assessment in Health Care, 2002. **18**(1): p. 46-54.
28. Gardiner, C., et al., *Methodological considerations for researching the financial costs of family caregiving within a palliative care context.* BMJ supportive & palliative care, 2016. **6**(4): p. 445-451.
29. May, P.N., C.: Morrison, R. S., *Economic impact of hospital inpatient palliative care consultation: review of current evidence and directions for future research.* Journal of Palliative Medicine, 2014. **17**(9): p. 1054-63.
30. Moher, D., et al., *Preferred reporting items for systematic review and meta-analysis protocols (PRISMA-P) 2015 statement.* Systematic reviews, 2015. **4**(1): p. 1.
31. Beller, E.M., et al., *PRISMA for abstracts: reporting systematic reviews in journal and conference abstracts.* PLoS medicine, 2013. **10**(4): p. e1001419.
32. van Mastrigt, G.A., et al., *How to prepare a systematic review of economic evaluations for informing evidence-based healthcare decisions: a five-step approach (part 1/3).* Expert review of pharmacoeconomics & outcomes research, 2016. **16**(6): p. 689-704.
33. Dy, S.M., et al., *Closing the quality gap: revisiting the state of the science (vol. 8: improving health care and palliative care for advanced and serious illness).* Evidence report/technology assessment, 2012(2088): p. 1.
34. Rietjens, J.A., et al., *Development and validation of search filters to find articles on palliative care in bibliographic databases.* Palliative medicine, 2019. **33**(4): p. 470-474.
35. Starr, L.T., et al., *Associations Among End-of-Life Discussions, Health-Care Utilization, and Costs in Persons With Advanced Cancer: A Systematic Review.* American Journal of Hospice and Palliative Medicine®, 2019: p. 1049909119848148.
36. Sassi, F., L. Archard, and D. McDaid, *Searching literature databases for health care economic evaluations: how systematic can we afford to be?* Medical care, 2002. **40**(5): p. 387-394.
37. McKinlay, R.J., N.L. Wilczynski, and R.B. Haynes, *Optimal search strategies for detecting cost and economic studies in EMBASE.* BMC Health Services Research, 2006. **6**(1): p. 67.
38. Gomes, B., et al., *Effectiveness and cost‐effectiveness of home palliative care services for adults with advanced illness and their caregivers.* Cochrane Database of Systematic Reviews, 2013(6).
39. Organization", W.H. *Definition of key terms* 2020 07.01.2020]; Available from: https://www.who.int/hiv/pub/guidelines/arv2013/intro/keyterms/en/.
40. Ryan, R., *Cochrane Consumers and Communication Review Group: data synthesis and analysis.* Cochrane Consumers and Communication Review Group, available at: http://cccrg. cochrane. org [Accessed 8 March 2016].[Google Scholar], 2013.
41. Husereau, D., et al., *Consolidated health economic evaluation reporting standards (CHEERS)—explanation and elaboration: a report of the ISPOR health economic evaluation publication guidelines good reporting practices task force.* Value in Health, 2013. **16**(2): p. 231-250.
42. Wijnen, B.F., et al., *How to prepare a systematic review of economic evaluations for informing evidence-based healthcare decisions: data extraction, risk of bias, and transferability (part 3/3).* Expert review of pharmacoeconomics & outcomes research, 2016. **16**(6): p. 723-732.

For peer review only - http://bmjopen.bmj.com/site/about/guidelines.xhtml

43. Excellence, N.-N.I.f.H.a.C. *Methods for the development of NICE public health guidance (third edition)*. 2012 accessed 07.01.2020]; Available from: https://www.nice.org.uk/process/pmg4/chapter/appendix-f-quality-appraisal-checklist-quantitative-intervention-studies.
44. Moher, D., et al., *Preferred reporting items for systematic reviews and meta-analyses: the PRISMA statement.* Annals of internal medicine, 2009. **151**(4): p. 264-269.
45. Puts, M.T.E., et al., *Clinical and Cost-effectiveness of a Comprehensive geriatric assessment and management for Canadian elders with Cancer-the 5C study: a study protocol for a randomised controlled phase III trial.* BMJ Open, 2019. **9**(5): p. e024485.

AUTHORS' CONTRIBUTIONS

JS and CF conceived of the idea, developed the research question, study methods and data extraction form. EC and CF designed and developed the search strategy and strings. CF drafted the manuscript with contribution from JS. All authors approved the final manuscript.

FUNDING STATEMENT

This systematic review is part of the 'iLIVE project –live well, die well, a research programme to support living until the end` which has received funding from the European Union's Horizon 2020 research and innovation programme under grant agreement No 825731. The funders did not play a role in decisions in the development or publishing of the protocol.

PATIENT CONSENT FOR PUBLICATION

Not required.

COMPETING INTERESTS STATEMENT

The authors declare that they have no conflict of interest.

For peer review only

For peer review only - http://bmjopen.bmj.com/site/about/guidelines.xhtml

APPENDIX

Appendix 1

PRISMA-P (Preferred Reporting Items for Systematic Review and Meta-Analysis Protocols) 2015 checklist: recommended items to address in a systematic review protocol*

Section and topic	Item No	Checklist item	Reported
**ADMINISTRATIVE INFORMATION**			
Title:			
Identification	1a	Identify the report as a protocol of a systematic review	Title page
Update	1b	If the protocol is for an update of a previous systematic review, identify as such	N/A
Registration	2	If registered, provide the name of the registry (such as PROSPERO) and registration number	Page 2
Authors:			
Contact	3a	Provide name, institutional affiliation, e-mail address of all protocol authors; provide physical mailing address of corresponding author	Title page
Contributions	3b	Describe contributions of protocol authors and identify the guarantor of the review	Page 10
Amendments	4	If the protocol represents an amendment of a previously completed or published protocol, identify as such and list changes; otherwise, state plan for documenting important protocol amendments	N/A
Support:			
Sources	5a	Indicate sources of financial or other support for the review	Page 10
Sponsor	5b	Provide name for the review funder and/or sponsor	Page 10
Role of sponsor or funder	5c	Describe roles of funder(s), sponsor(s), and/or institution(s), if any, in developing the protocol	Page 10
**INTRODUCTION**			
Rationale	6	Describe the rationale for the review in the context of what is already known	Page 3
Objectives	7	Provide an explicit statement of the question(s) the review will address with reference to participants, interventions, comparators, and outcomes (PICO)	Page 3-4
**METHODS**			
Eligibility criteria	8	Specify the study characteristics (such as PICO, study design, setting, time frame) and report characteristics (such as years considered, language, publication status) to be used as criteria for eligibility for the review	Page 4-6

For peer review only

Information sources	9	Describe all intended information sources (such as electronic databases, contact with study authors, trial registers or other grey literature sources) with planned dates of coverage	Page 5
Search strategy	10	Present draft of search strategy to be used for at least one electronic database, including planned limits, such that it could be repeated	Page 6-7 Appendix 2
Study records:			
Data management	11a	Describe the mechanism(s) that will be used to manage records and data throughout the review	Page 6
Selection process	11b	State the process that will be used for selecting studies (such as two independent reviewers) through each phase of the review (that is, screening, eligibility and inclusion in meta-analysis)	Page 7-8
Data collection process	11c	Describe planned method of extracting data from reports (such as piloting forms, done independently, in duplicate), any processes for obtaining and confirming data from investigators	Page 8
Data items	12	List and define all variables for which data will be sought (such as PICO items, funding sources), any pre-planned data assumptions and simplifications	Page 4-6
Outcomes and prioritization	13	List and define all outcomes for which data will be sought, including prioritization of main and additional outcomes, with rationale	Page 4 and 6
Risk of bias in individual studies	14	Describe anticipated methods for assessing risk of bias of individual studies, including whether this will be done at the outcome or study level, or both; state how this information will be used in data synthesis	Page 9
Data synthesis	15a	Describe criteria under which study data will be quantitatively synthesised	Page 8
	15b	If data are appropriate for quantitative synthesis, describe planned summary measures, methods of handling data and methods of combining data from studies, including any planned exploration of consistency (such as I^2, Kendall's τ)	N/A
	15c	Describe any proposed additional analyses (such as sensitivity or subgroup analyses, meta-regression)	N/A
	15d	If quantitative synthesis is not appropriate, describe the type of summary planned	N/A
Meta-bias(es)	16	Specify any planned assessment of meta-bias(es) (such as publication bias across studies, selective reporting within studies)	N/A
Confidence in cumulative evidence	17	Describe how the strength of the body of evidence will be assessed (such as GRADE)	Page 9

* It is strongly recommended that this checklist be read in conjunction with the PRISMA-P Explanation and Elaboration (cite when available) for important clarification on the items. Amendments to a review protocol should be tracked and dated. The copyright for PRISMA-P (including checklist) is held by the PRISMA-P Group and is distributed under a Creative Commons Attribution Licence 4.0.

For peer review only

The PRISMA for Abstracts Checklist

TITLE	CHECKLIST ITEM	REPORTED ON PAGE #
**1.Title**	Identify the report as a systematic review, meta-analysis, or both.	2 (Introduction)
**BACKGROUND**		
**2.Objectives:**	The research question including components such as participants, interventions, comparators, and outcomes.	2 (Introduction)
**METHODS**		
**3.Eligibility criteria:**	Study and report characteristics used as criteria for inclusion.	2 (Introduction)
**4.Information sources:**	Key databases searched and search dates.	2 (Methods and analysis)
**5. Risk of bias:**	Methods of assessing risk of bias.	2 (Methods and analysis)
**RESULTS**		
**6. Included studies:**	Number and type of included studies and participants and relevant characteristics of studies.	n.a.
**7. Synthesis of results:**	Results for main outcomes (benefits and harms), preferably indicating the number of studies and participants for each. If meta-analysis was done, include summary measures and confidence intervals.	n.a.
**8. Description of the effect:**	Direction of the effect (i.e. which group is favoured) and size of the effect in terms meaningful to clinicians and patients.	n.a.
**DISCUSSION**		
**9. Strengths and Limitations of evidence:**	Brief summary of strengths and limitations of evidence (e.g. inconsistency, imprecision, indirectness, or risk of bias, other supporting or conflicting evidence)	n.a.
**10. Interpretation:**	General interpretation of the results and important implications	n.a.
**OTHER**		
**11. Funding:**	Primary source of funding for the review.	2 (Acknowledgements)
**12. Registration:**	Registration number and registry name.	2 (Methods and analysis)

Appendix 2

Search syntax in different databases

EMBASE.com

#33 #28 NOT #31 AND [1-1-1999]/sd NOT [15-11-2019]

#32 #28 NOT #31

#31 #29 NOT #30

#30 #29 AND ([young adult]/lim OR [adult]/lim OR [middle aged]/lim OR [aged]/lim OR [very elderly]/lim)

#29 #28 AND ([adolescent]/lim OR [child]/lim OR [fetus]/lim OR [infant]/lim OR [newborn]/lim OR [preschool]/lim OR [school]/lim)

#23 NOT #26 AND ([dutch]/lim OR [english]/lim OR [french]/lim OR [german]/lim OR

#28 [spanish]/lim)

#27 #23 NOT #26

#26 #24 OR #25

#25 waste:jt OR 'life cycle assessment':jt

#24 recycl*:ti,de,kw,jt

#23 #13 AND #22

#22 #14 OR #15 OR #16 OR #17 OR #18 OR #19 OR #20 OR #21

#21 (('health care' OR healthcare) NEAR/3 cost*):ti,kw

#20 economics:ti,kw

(economic* NEAR/3 (evaluat* OR aspect* OR health OR analy* OR model* OR framework* OR

#19 'frame work*' OR method*)):ti,ab,kw

#18 'health care cost'/exp/mj

#17 'economics'/mj

#16 'economic model'/de

#15 'health economics'/de

For peer review only

#14 'economic evaluation'/de

#13 #1 OR #2 OR #3 OR #4 OR #5 OR #6 OR #7 OR #8 OR #9 OR #10 OR #11 OR #12

#12 bereave*:de,ti,ab,kw

#11 hospice*:de,ti,ab,kw

#10 'advanced cancer':ti,ab,kw

#9 'end of life':de,ti,ab,kw OR 'last year of life':de,ti,ab,kw OR 'lyol':de,ti,ab,kw OR 'life s end':de,ti,ab,kw

#8 (terminal* NEAR/6 (care* OR caring OR ill OR illness* OR patient*)):ti,ab,kw

#7 palliat*:ti,ab,kw

#6 'hospice'/de

#5 'terminal disease'/de

#4 'palliative therapy'/exp

#3 'terminal care'/exp

#2 'terminally ill patient'/exp

#1 'palliative nursing'/de

EBM Reviews - Health Technology Assessment Database (OVID) (discontinued at the end of 2016)

1 Palliative Care/

2 exp Terminal Care/

3 terminally ill/

4 palliat*.mp.

5 (terminal* adj6 (care or caring or ill or illness*)).mp.

6 (end of life or last year of life or lyol or life* end).mp.

7 advanced cancer.mp.

8 hospices/

9 hospice*.mp.

For peer review only

10 bereave*.mp.

11 1 or 2 or 3 or 4 or 5 or 6 or 7 or 8 or 9 or 10

12 exp Health Care Costs/

13 ((health care or healthcare) adj3 cost*).mp.

14 "Costs and Cost Analysis"/

15 Cost-Benefit Analysis/mt [Methods]

16 exp models, economic/

17 (economic* adj3 (evaluat* or aspect* or health or analy* or model* or framework* or frame work* or method*)).mp.

18 economics.mp.

19 12 or 13 or 14 or 15 or 16 or 17 or 18

20 11 and 19

21 limit 20 to "all child (0 to 18 years)"

22 limit 20 to (dutch or english or german or spanish or french)

EBM Reviews - NHS Economic Evaluation Database (OVID) (discontinued at the 31st March 2015)

1 Palliative Care/

2 exp Terminal Care/

3 terminally ill/

4 palliat*.mp. [mp=title, text, subject heading word]

5 (terminal* adj6 (care or caring or ill or illness*)).mp. [mp=title, text, subject heading word]

6 (end of life or last year of life or lyol or life* end).mp. [mp=title, text, subject heading word]

7 advanced cancer.mp. [mp=title, text, subject heading word]

8 hospices/

9 hospice*.mp. [mp=title, text, subject heading word]

10 bereave*.mp. [mp=title, text, subject heading word]

11 1 or 2 or 3 or 4 or 5 or 6 or 7 or 8 or 9 or 10

For peer review only

12	limit 11 to "all child (0 to 18 years)"
13	limit 12 to "all adult (19 plus years)"
14	12 not 13
15	11 not 14
16	limit 15 to yr="1999 - 2015"

For peer review only

Appendix 3

Data extraction form: methodological studies

**Reviewer:**	....	**Date:**	....
**Author:**	....	**Country:**	....
**Reference type:**	....	**Manuscript code:**	....
**Name of journal/book chapter/website etc. :**	....	**Language of publication**	....
**Date of publication:**	....	**Page number:**	....
**Study design:**			
Case report	O	Review (scoping, systematic, narrative)	O
Economic evaluation	O	Guideline or checklist	O
Editorial/commentary/letter:	O	Other	O
Cohort	O		
**Quality appraisal score:**			
Quality appraisal applied:	....	Quality rating:	....
**Aspect of economic evaluation (EE) described:**			
O Study population	O Study perspective	O Time horizon	O Type of EE
O Outcomes	O Data (sources)	O Costs	O Resource use measurement
O Valuation method	O Adjustments for inflation/deflation	O Discounting	O Ethical implications
O Decision modeling	O Care setting	O Other:	

For peer review only

Data extraction form: applied economic evaluations

**Reviewer:**	…………………………	**Date:**	…………………………
**Author:**	…………………………	**Manuscript code:**	…………………………
**Name of journal:**	…………………………	**Language of publication**	…………………………
**Date of publication:**	…………………………		
**Study characteristics:**			
Funding source (p.n.*)	…………………………	Year of analysis (p.n.):	…………………………
Study design (p.n.):	…………………………	Type of economic evaluation (CEA, CUA., etc) (p.n.):	…………………………
Author stated analytical perspective (p.n.):	…………………………	Time frame of analysis (p.n.):	…………………………
Country (p.n.):	…………………………	Study sector (hospice, home, hospital, etc. (p.n.):	…………………………
Modeling used (p.n.):	…………………………	Quality appraisal score (Quality appraisal):	…………………………
Intervention description:	…………………………		
**Patient cohort characteristics:**			
Control group size:	…………………………	Intervention group size:	…………………………
Target population definiton:	…………………………		
**Methods :**			
Analysis of uncertainty:	…………………………		
**Outcomes :**			
Outcomes measured:	…………………………	Discounting:	…………………………
Data sources :	…………………………		
**Costs:**			
Cost categories included:	…………………………	Discounting:	…………………………

For peer review only

Unit cost sources:	…………………………….	Resource use info source:	…………………………….
Reviewer stated analytical perspective:	…………………………….	Year of costing:	…………………………….
Adjustments for inflation/deflation:	…………………………….		
**Cost-effectiveness:**			
Main CE results:	…………………………….	Analysis of uncertainty:	…………………………….

*p.n. - page number

For peer review only

PRISMA-P (Preferred Reporting Items for Systematic review and Meta-Analysis Protocols) 2015 checklist: recommended items to address in a systematic review protocol*

Section and topic	Item No	Checklist item	Reported
**ADMINISTRATIVE INFORMATION**			
Title:			
Identification	1a	Identify the report as a protocol of a systematic review	Title page
Update	1b	If the protocol is for an update of a previous systematic review, identify as such	N/A
Registration	2	If registered, provide the name of the registry (such as PROSPERO) and registration number	Page 2
Authors:			
Contact	3a	Provide name, institutional affiliation, e-mail address of all protocol authors; provide physical mailing address of corresponding author	Title page
Contributions	3b	Describe contributions of protocol authors and identify the guarantor of the review	Page 10
Amendments	4	If the protocol represents an amendment of a previously completed or published protocol, identify as such and list changes; otherwise, state plan for documenting important protocol amendments	N/A
Support:			
Sources	5a	Indicate sources of financial or other support for the review	Page 10
Sponsor	5b	Provide name for the review funder and/or sponsor	Page 10
Role of sponsor or funder	5c	Describe roles of funder(s), sponsor(s), and/or institution(s), if any, in developing the protocol	Page 10
**INTRODUCTION**			
Rationale	6	Describe the rationale for the review in the context of what is already known	Page 3
Objectives	7	Provide an explicit statement of the question(s) the review will address with reference to participants, interventions, comparators, and outcomes (PICO)	Page 3-4
**METHODS**			
Eligibility criteria	8	Specify the study characteristics (such as PICO, study design, setting, time frame) and report characteristics (such as years considered, language, publication status) to be used as criteria for eligibility for the review	Page 4-6
Information sources	9	Describe all intended information sources (such as electronic databases, contact with study authors, trial registers or other grey literature sources) with planned dates of coverage	Page 5
Search strategy	10	Present draft of search strategy to be used for at least one electronic database, including planned limits, such that it could be repeated	Page 6-7<br>Appendix 2

For peer review only

Study records:			
Data management	11a	Describe the mechanism(s) that will be used to manage records and data throughout the review	Page 6
Selection process	11b	State the process that will be used for selecting studies (such as two independent reviewers) through each phase of the review (that is, screening, eligibility and inclusion in meta-analysis)	Page 7-8
Data collection process	11c	Describe planned method of extracting data from reports (such as piloting forms, done independently, in duplicate), any processes for obtaining and confirming data from investigators	Page 8
Data items	12	List and define all variables for which data will be sought (such as PICO items, funding sources), any pre-planned data assumptions and simplifications	Page 4-6
Outcomes and prioritization	13	List and define all outcomes for which data will be sought, including prioritization of main and additional outcomes, with rationale	Page 4 and 6
Risk of bias in individual studies	14	Describe anticipated methods for assessing risk of bias of individual studies, including whether this will be done at the outcome or study level, or both; state how this information will be used in data synthesis	Page 9
Data synthesis	15a	Describe criteria under which study data will be quantitatively synthesised	Page 8
	15b	If data are appropriate for quantitative synthesis, describe planned summary measures, methods of handling data and methods of combining data from studies, including any planned exploration of consistency (such as I^2, Kendall's τ)	N/A
	15c	Describe any proposed additional analyses (such as sensitivity or subgroup analyses, meta-regression)	N/A
	15d	If quantitative synthesis is not appropriate, describe the type of summary planned	N/A
Meta-bias(es)	16	Specify any planned assessment of meta-bias(es) (such as publication bias across studies, selective reporting within studies)	N/A
Confidence in cumulative evidence	17	Describe how the strength of the body of evidence will be assessed (such as GRADE)	Page 9

* It is strongly recommended that this checklist be read in conjunction with the PRISMA-P Explanation and Elaboration (cite when available) for important clarification on the items. Amendments to a review protocol should be tracked and dated. The copyright for PRISMA-P (including checklist) is held by the PRISMA-P Group and is distributed under a Creative Commons Attribution Licence 4.0.

For peer review only
